# Use of the Novel Site-Directed Enzyme Enhancement Therapy (SEE-Tx) Drug Discovery Platform to Identify Pharmacological Chaperones for Glutaric Acidemia Type 1

**DOI:** 10.1021/acs.jmedchem.4c00292

**Published:** 2024-09-23

**Authors:** Madalena Barroso, Alexandra Puchwein-Schwepcke, Lars Buettner, Ingrid Goebel, Katrin Küchler, Ania C. Muntau, Aida Delgado, Ana M. Garcia-Collazo, Marc Martinell, Xavier Barril, Elena Cubero, Søren W. Gersting

**Affiliations:** † University Children’s Research, UCR@Kinder-UKE, University Medical Center Hamburg-Eppendorf, Hamburg 20246, Germany; ‡ Department of Molecular Pediatrics, Dr. von Hauner Children’s Hospital, Ludwig-Maximilians-University, Munich 80337, Germany; § Department of Pediatric Neurology and Developmental Medicine, University Children’s Hospital Basel, UKBB, Basel 4031, Switzerland; ∥ Pharmaceutical Development Biologicals, Boehringer Ingelheim Pharma GmbH & Co. KG, Biberach an der Riss 88397, Germany; ⊥ University Children’s Hospital, University Medical Center Hamburg-Eppendorf, Hamburg 20246, Germany; # German Center for Child and Adolescent Health (DZKJ), Partner Site Hamburg, University Medical Center Hamburg-Eppendorf, Hamburg 20246, Germany; ∇ Gain Therapeutics Sucursal en España, Parc Científic de Barcelona, Barcelona 08028, Spain; ○ Minoryx Therapeutics S.L., Tecno Campus Mataró-Maresme, Mataró, Barcelona 08302, Spain

## Abstract

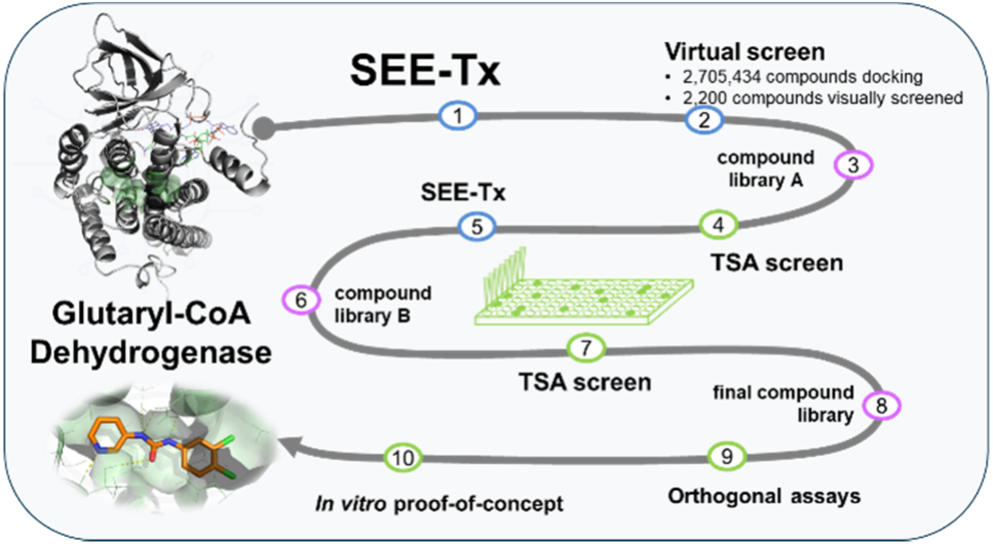

Allosteric regulators acting as pharmacological chaperones hold promise for innovative therapeutics since they target noncatalytic sites and stabilize the folded protein without competing with the natural substrate, resulting in a net gain of function. Exogenous allosteric regulators are typically more selective than active site inhibitors and can be more potent than competitive inhibitors when the natural substrate levels are high. To identify novel structure-targeted allosteric regulators (STARs) that bind to and stabilize the mitochondrial enzyme glutaryl-CoA dehydrogenase (GCDH), the computational site-directed enzyme enhancement therapy (SEE-Tx) technology was applied. SEE-Tx is an innovative drug discovery platform with the potential to identify drugs for treating protein misfolding disorders, such as glutaric acidemia type 1 (GA1) disease. Putative allosteric regulators were discovered using structure- and ligand-based virtual screening methods and validated using orthogonal biophysical and biochemical assays. The computational approach presented here could be used to discover allosteric regulators of other protein misfolding disorders.

## Introduction

Glutaric acidemia type 1 (GA1, OMIM #231670) is a rare metabolic disorder caused by an inherited deficiency of the mitochondrial enzyme glutaryl-CoA dehydrogenase (GCDH), with an estimated prevalence ranging from 1 in 125,000 to 1 in 250,000 in newborns within high-risk populations.^1^ The clinical course of GA1 usually features an episode of acute metabolic encephalopathy, which results in an irreversible striatal injury in more than 80% of untreated children.^2^ Current treatments include dietary intervention (low-lysine and tryptophan-reduced diet), carnitine supplementation, and emergency treatment with intravenous dextrose, saline, and l-carnitine during a fever or acute illness.^3,4^ However, despite the burdensome dietary and pharmacological treatments, a considerable percentage of GA1 patients (approximately 15%) still experience devastating neurological complications.^1,5^ Over 200 pathogenic variants of the GCDH gene have been reported, with about 60% being missense variants.^6^ Several of these GCDH missense variants have been characterized and helped to define GA1 as a protein misfolding disorder.^7,8^ GCDH naturally exists as a homotetramer comprising four identical subunits that assemble into a functional enzyme complex. This quaternary structure is critical for its enzymatic activity, as it facilitates the proper alignment of the active sites and the necessary conformational changes necessary for catalysis. Misfolding and destabilization of the protein tetramer, leading to functional deficiency of GCDH has been associated with several missense variants, such as V400M and others.^7,8^ Therefore, misfolding prevention of GCDH proteins by pharmacological chaperone therapy (PCT) is a promising approach for GA1.^8,9^ Pharmacological chaperones are small molecules that bind and stabilize misfolded proteins, rescuing their function. Considerable success has been achieved in recent years with pharmacological chaperones in either correcting misfolding or stabilizing active but unstable proteins. This strategy is being applied in clinical practice and is transforming the treatment of protein misfolding diseases with a loss-of-protein-function phenotype, such as cystic fibrosis, lysosomal storage disorders, and phenylketonuria.^10−15^ To date, the majority of pharmacological chaperones bind to the active site of their target enzyme, acting as reversible competitive inhibitors.^10,16^ Although the stabilizing activity of the pharmacological chaperone is necessary, its inhibitory activity is neither needed nor desirable.^17^ Allosteric regulators that interact with noncatalytic regions of the target enzyme may represent an alternative PCT strategy,^18^ which could afford safer daily dose regimens by avoiding the use of active site binders with a potential inhibitory effect.^16,17^ As allosteric sites are evolutionary and less conserved, they may also yield more selective compounds.^19^

The computational site-directed enzyme enhancement therapy (SEE-Tx) technology is an innovative drug discovery platform for the identification of structure-targeted allosteric regulator (STAR) molecules.^20,21^ SEE-Tx combines multiple structure-based computational methods and chemoinformatic tools to identify druggable allosteric sites and molecules that bind to them. The broad application potential of this computational drug discovery platform was recently demonstrated with the discovery of a first-in-class allosteric regulator targeting the lysosomal enzyme β-glucocerebrosidase (GCase), mutations of which are causal for the rare autosomal storage disorder Gaucher disease.^22,23^ To search for putative allosteric molecules for conformational stabilization of misfolded GCDH, we performed druggability and virtual screening studies using the SEE-Tx computational platform, leading to the identification of novel allosteric compounds that bind to and stabilize GCDH with the potential to enter the development process as pharmacological chaperones of GA1.

## Results

### Identification of Allosteric Binding Sites

**Figure 1:**
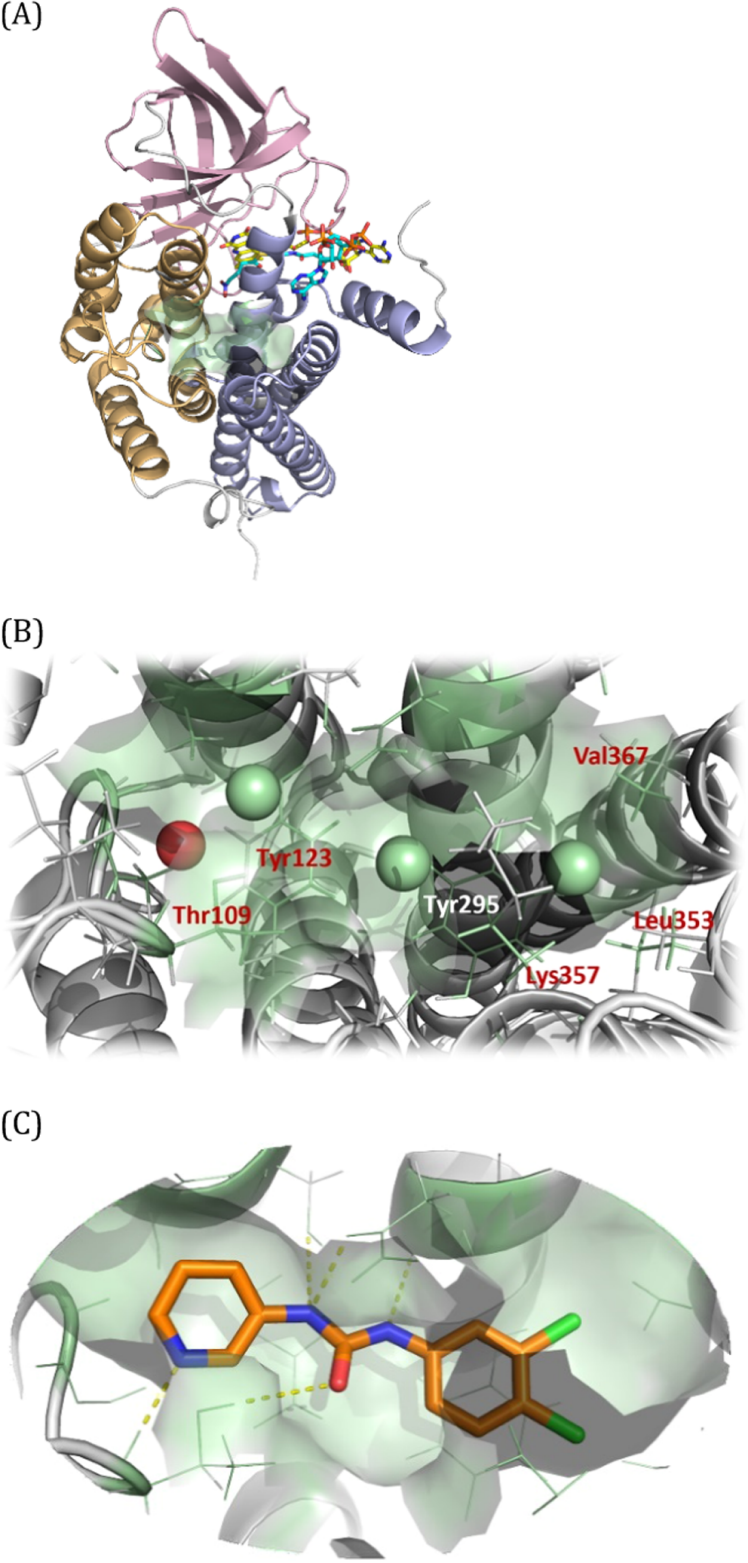
Putative allosteric binding site of glutaryl-CoA dehydrogenase (GCDH) monomer identified using the SEE-Tx platform. (A) Overview of GCDH structure (1SIR.pdb) showing the allosteric binding site (green area) at the interface of the α-helical bundle amino-terminal domain (light orange) and the α-helical domain at the carboxyl terminus (light blue). The active site is located at the opposite side of the protein, at a distance of 12 Å. S-4-nitrobutyryl-CoA (an alternative substrate) and FAD (cofactor) are shown as cyan and yellow sticks, respectively. (B) MDmix identified binding hot spots at the allosteric site corresponding to polar (red) and lipophilic (green) interactions. (C) Predicted binding mode of compound A19 within the allosteric pocket. H-bonding interactions are indicated by yellow dashed lines.

A key component of SEE-Tx is the application of the MDmix methodology^24−27^ with proprietary energetic corrections^28,29^ to quantitatively identify binding hot spots on the protein surface, which can be clustered into druggable binding sites.^24^ We applied the method to the published GCDH three-dimensional structure (1SIQ.pdb)^30^ which revealed a druggable allosteric site in each of the protein’s four monomers. The identified allosteric binding site comprises 18 residues: Gly107, Pro108, Thr109, Ile110, Ser120, Tyr123, Ser145, Ser146, Met149, His150, Asn291, Asn292, Tyr295, Leu353, Gly354, Lys357, Ala362, and Val367 (following the GCDH full-length protein sequence). These sites are located at a region where the two α-helical bundle domains of the GCDH protein converge, at the back of the active site (Figure 1A). The allosteric and active sites in each monomer are approximately 12 Å apart, suggesting distinct but potentially interactive functional areas. The substrate binding cavity is tubular and narrow. Most of the substrate is buried inside the monomer’s structure, while the adenosine diphosphate moiety of the FAD cofactor remains exposed to the solvent and proximal to the allosteric site identified in this study. Given its location, it is plausible that a molecule binding to the identified allosteric site could not only stabilize the native 3D conformation of GCDH but also influence the substrate and the catalytic performance of the protein. This hypothesis is further substantiated by the fact that 10 of the 18 residues defining our allosteric binding site were identified as pathogenic missense variants of GCDH.^31^ These variants are associated with a loss of enzymatic activity in GA1 patients, underscoring the critical role of these regions in the protein’s function.^31^

The main interaction hot spots identified by MDmix correspond to hydrogen bonds with the backbone (N) of Thr109. Other preferred binding hot spots were three hydrophobic interactions with the side chains of Tyr123, Tyr295, Lys357, Leu353, and Val367 (Figure 1B). The presence of four strong binding hot spots in close proximity reveals that the allosteric binding site can display substantial binding affinity for small drug-like ligands. These hot spots were used to define pharmacophoric points (one hydrogen bond acceptor plus three hydrophobic groups) and were used as restraints in docking-based virtual screening. Figure 1C displays a predicted binding mode of an example compound (A19) within the predicted allosteric pocket, showing the hydrogen bond interactions established.

### Virtual Screening and Experimental Validation Strategy

**Figure 2:**
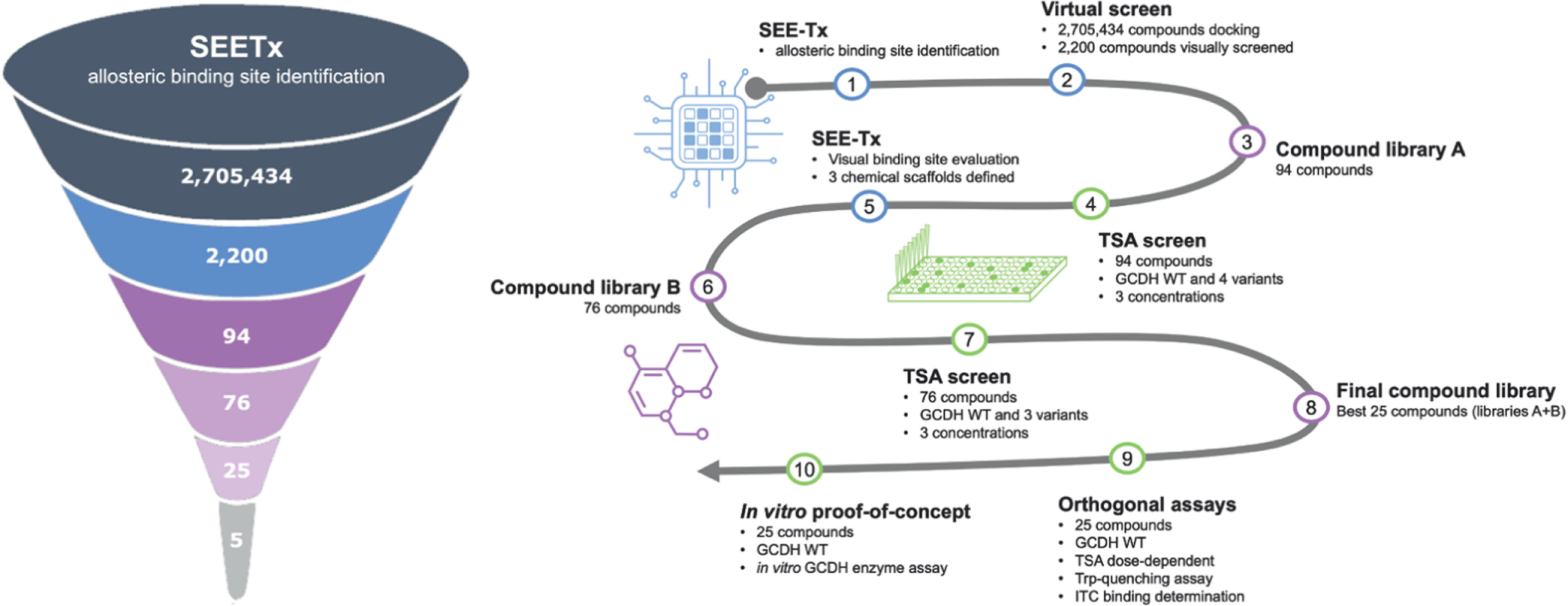
Drug discovery platform applied for the identification of potential pharmacological chaperones for glutaric acidemia type 1. SEE-Tx methodology uses a structure-based approach to identify putative binders for glutaryl-CoA dehydrogenase (GCDH). This was followed by a step-by-step validation approach using thermal shift assays and additional orthogonal biophysical and biochemical assays, which allowed the identification of 5 validated allosteric regulators of GCDH.

Starting from nearly three million input molecules, a pharmacophoric-restrained docking protocol (see the Experimental Section) identified 94 molecules (Library A) that were subsequently purchased and tested by a thermal shift assay (TSA), resulting in several hit compounds. Using the validated experimental hits as reference, we performed a SAR-by-catalogue search, generating a set of hit analogues that were again purchased and screened by TSA (76 compounds; Library B). The compounds were selected based on their molecular weight, lipophilicity (log *P*), structural features, drug-likeness rules (HBA, HBD, Rot. Bonds, TPSA), reactive compounds (REOS), frequent hitters (PAINS), and data resulting from virtual screening (molecular docking). Following experimental validation, the most promising molecules from the hit lists of screens A and B were combined, resulting in a prioritized hit series for the GCDH target. Subsequently, a final list of 25 promising compounds was selected for further characterization (Figure 2).

### Primary Screening Using Thermal Stability Assays

**Figure 3:**
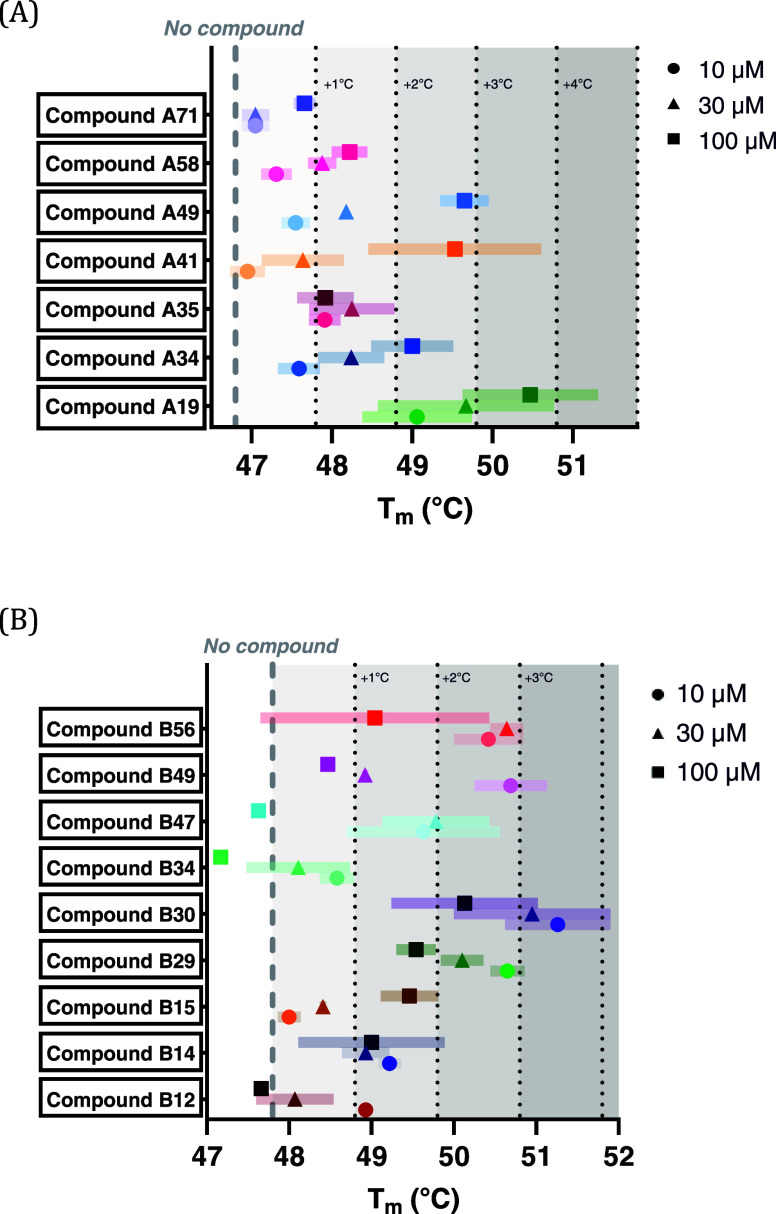
Dose-dependent effect on thermal stability of glutaryl-CoA dehydrogenase (GCDH) wild type (WT) in the presence of (A) compounds A19, A34, A35, A41, A49, A58 and A71 from library A, and (B) compounds B12, B14, B15, B29, B30, B34, B47, B49, and B56 from library B, at three different concentrations (10, 30, and 100 μM). Most compounds have a shift in Tm (melting temperature: the temperature at which 50% of a protein is unfolded) relative to the baseline (“No compound,” dotted line), suggesting protein stabilization. *T*_m_ for each compound concentration is shown (circle, triangle, square, for 10, 30, and 100 μM, respectively) ± standard deviation (transparent bar behind Tm value). Gray shaded blocks represent Δ*T*_m_ shifts of >1 °C, >2 °C and >3 °C.

Using a two-step biophysical screening approach, TSA was used to monitor the thermal denaturation of GCDH wild type (WT) and four variants (R88C, V400M, E414 K, and A433E) in the absence and presence of each of the 94 compounds from library A (Table S3). GCDH variants R88C, V400M, E414 K, and A433E for the TSA-based primary screening were chosen because of their documented prevalence among GCDH mutations, their differential residual enzymatic activities, protein sequence, and domain location.^7^ First, TSA was conducted for each compound at three different concentrations (10, 30, and 100 μM) to identify specific binders positively affecting the thermal stability of both WT and variant GCDH proteins. Positive hits were ranked based on their TSA stabilizing effects (scores of 1 for Δ*T*_m_ > 1 °C, 2 for Δ*T*_m_ > 2 °C and 4 for Δ*T*_m_ > 3 °C), with the highest Δ*T*_m_ from three tested concentrations determining each compound’s score. Scores from the wild type and four variants were then combined (summed) for each compound and used for the final ranking and hit selection. Following the initial TSA screening, 7 out of the 13 best primary hits can be classified into two chemical scaffolds and the remaining were structurally diverse (Table 1). The top seven compounds (A19, A34, A35, A41, A49, A58, and A71) were further validated using TSA, presenting a dose-dependent effect on thermal stability for GCDH WT (Figure 3A). Subsequently, a second compound library of 76 analogue compounds was designed for further screening (library B; see Table S4), resulting in the identification of 9 standout hits (Figure 3B). Compound B15 demonstrates a clear dose–response relationship, while other compounds, such as B14, present consistent Δ*T*m values across all three concentrations, which could suggest that their stabilizing effects plateau or saturate at lower concentrations. Compounds B29, B30, B47, B49, and B56 stand out as effective stabilizers, with higher effects at the lowest concentrations tested (≥2 °C). The absence of a clear dose-dependent effect for the WT protein might be indicative of insolubility at higher concentrations. Overall, no direct correlation between the compounds’ solubility and the combined effects in thermal stability could be found, except for the highest concentration tested during the screening, 100 μM (see Figure S2).

**Table 1:** Summary of the Effects of the Final 25-Compound Library on GCDH^a^

			stability effect (Δ*T*_m_ score)					
compound	scaffold	relative enzymatic activity (% DMSO)	WT	R88C	V400M	A433E	Trp quenching (*K*_B_; μM)	ITC (*K*_d_; μM)
A19	1	122 ± 20	2	4	4	4	283	insoluble
A25	1	117 ± 7	2	2	0	2	182	no binding
A29	1	141 ± 11	1	2	0	0	133	no binding
A34	2	127 ± 4	4	4	0	2	178	insoluble
A35	2	104 ± 25	4	4	0	4	332	no binding
A41	1	135 ± 59	4	2	0	4	428	8.2 ± 4.5
A49	others	48 ± 12	0	2	4	1	55	3.4 ± 1.3
A52	others	124 ± 3	0	2	0	2	96	insoluble
A55	others	127 ± 7	1	2	0	2	46	insoluble
A57	others	132 ± 10	0	4	0	0	224	insoluble
A58	others	115 ± 3	0	4	4	0	307	6.9 ± 2.5
A66	others	81 ± 0	0	2	2	1	51	44.9 ± 7.0
A71	2	132 ± 5	0	4	4	2	92	7.7 ± 1.3
B12	others	115 ± 19	0	1	0	0	314	no binding
B14	2	111 ± 4	1	1	2	0	94	insoluble
B15	2	112 ± 0	2	1	4	1	85	no binding
B29	1	134 ± 4	2	2	4	2	65	insoluble
B30	1	124 ± 8	4	4	0	0	106	insoluble
B31	1	147 ± 5	4	1	1	1	58	insoluble
B34	2	152 ± 13	0	0	0	0	145	no binding
B37	others	140 ± 13	0	0	0	0	88	insoluble
B40	others	119 ± 10	4	0	–4	–4	44	insoluble
B47	1	122 ± 10	2	1	4	0	61	no binding
B49	others	2.6 ± 2.3	4	2	1	1	45	insoluble
B56	1	113 ± 5	4	4	4	0	103	insoluble

a Scaffold, scaffold group; GCDH wild type (150 nM) activity was determined in the presence of 10 μM compound (in comparison to DMSO control); Δ*T*m, change in melting temperature; Δ*T*_m_ score, assigned based on the first Δ*T*_m_ for each protein variant, with scores of 1 given for Δ*T*_m_ > 1 °C, 2 for Δ*T*_m_ > 2 °C and 4 for Δ*T*_m_ > 3 °C; Trp, tryptophan quenching; ITC, isothermal titration calorimetry; *K*_B_, binding affinity constant; *K*_d_, dissociation constant; GCDH, glutaryl-CoA dehydrogenase; WT, wild type.

Overall, 170 compounds from libraries A and B (Table S1) were screened following a two-step biophysical screening approach using TSA. All hits identified are small and nonsubstrate-like molecules with good optimization potential. A curated compound library was established, comprising 25 compounds from libraries A and B and spanning two chemical scaffolds and several promising singletons (Tables 1 and S5). In the secondary screening phase, this final compound library underwent comprehensive in vitro analyses, including dose–response TSA, tryptophan (Trp) quenching, enzymatic activity, and isothermal titration calorimetry (ITC) assays.

### Secondary Screening—Tryptophan Fluorescence Assays

**Figure 4:**
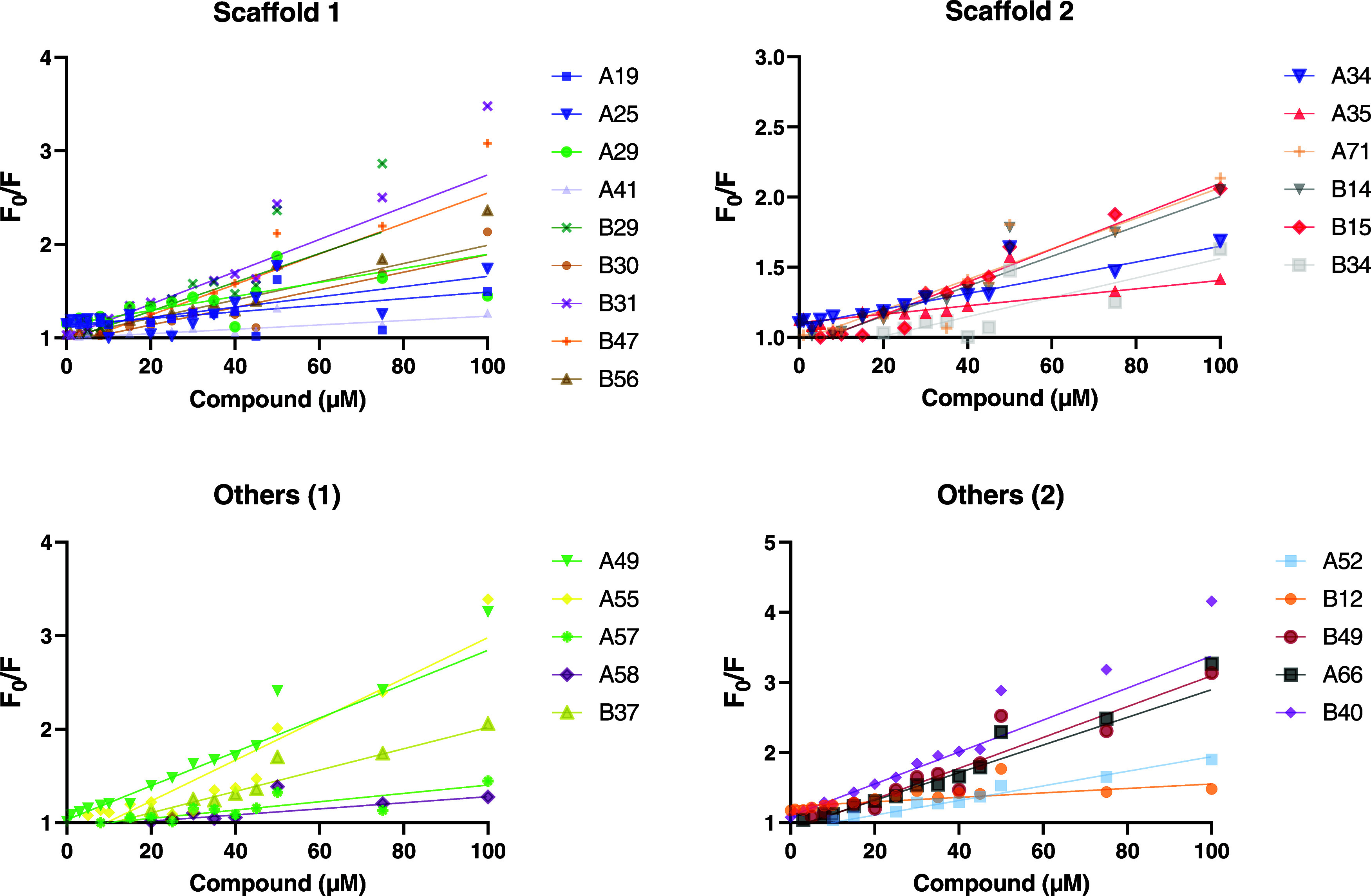
Tryptophan fluorescence quenching for glutaryl-CoA dehydrogenase (GCDH) wild type (WT) with increasing compound concentrations of the 25 selected compounds, comprising two main scaffolds and various singletons (“Others 1” and “Others 2”). Intrinsic fluorescence of GCDH WT is quenched due to the binding of compounds at increasing concentrations. *F*_0_, initial fluorescence intensity; *F*, fluorescence intensity in the presence of a quenching agent.

To further characterize the final hit compound library, tryptophan quenching assays were conducted to indirectly determine each compound’s binding affinity for GCDH. Figure 4 depicts a decrease in fluorescence intensity observed following the titration of the 25 compounds to GCDH WT, and the calculated slopes were used to define binding affinity constants (*K*_B_; corresponding to the inverse of the calculated titration slope). Among these, 13 exhibited a quenching effect, resulting in a binding affinity of less than 100 μM. This representation spans all tested scaffolds: 33% from scaffold 1 (3/9), 50% from scaffold 2 (3/6), and 70% from other scaffolds (7/10).

### Secondary Screening—Isothermal Titration Calorimetry

**Figure 5:**
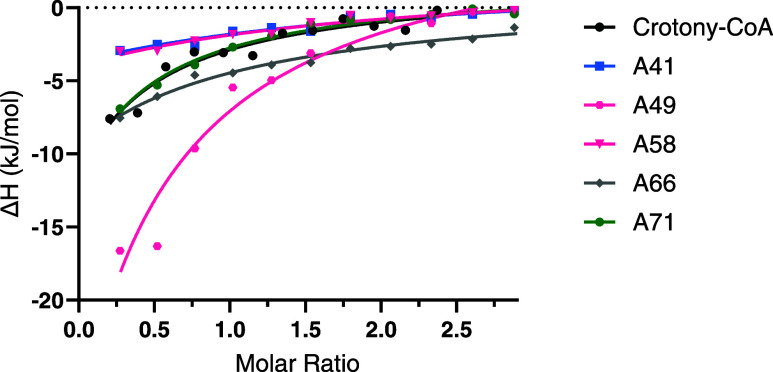
Isothermal titration calorimetry (ITC) analysis of the interaction of glutaryl-CoA dehydrogenase (GCDH) wild type (WT) with compounds A41, A49, A58, A66 and A71. Crotonyl-CoA, the GCDH enzyme reaction product, was used as a positive binding control. Δ*H* (change in enthalpy derived from integration of the heat peak intensities) is plotted against the GCDH WT/compound molar ratio (based on monomer concentration).

Isothermal titration calorimetry (ITC) is widely recognized as a gold standard to assess molecular interactions and determine binding affinities. We performed ITC to confirm the GCDH binding molecules, retrieving binding affinity and thermodynamic information on the specific protein–ligand interactions. Several compounds became insoluble upon dilution into protein buffer (final concentration: 5% DMSO), hindering the assessment of their binding through ITC. Table S6 summarizes the key parameters obtained from ITC experiments conducted with GCDH WT and the binding compounds A41, A49, A58, A66, and A71, which were soluble in aqueous buffer. In Figure 5, the respective ITC titration curves are shown. Four compounds exhibited a binding affinity (*K*_d_) within the single-digit micromolar range, whereas compound A66 was double-digit micromolar (*K*_d_ = 44 μM). Binding was driven by favorable (negative) binding enthalpy (Δ*H*) and entropy (−*T*Δ*S*) energies. Compound A49 presented the strongest binding to GCDH (*K*_d_ = 3.4 μM) and the associated thermodynamic binding profile suggests that enthalpic changes are the major driving force of this interaction.

### Secondary Screening—Enzyme Activity

**Figure 6:**
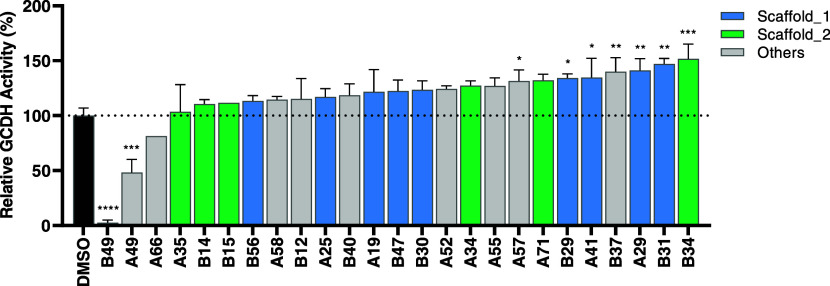
Effect of the final compound library on the catalytic activity of recombinant GCDH WT. The selected scaffolds are color-coded (scaffold 1 in blue; scaffold 2 in green; other individual scaffolds in gray). The mean values and standard deviations were determined from three independent assays (*, *p* < 0.05; **, *p* < 0.005; ***, *p* < 0.0005). DMSO, dimethyl sulfoxide; GCDH, glutaryl-CoA dehydrogenase; WT, wild type.

To assess the putative allosteric effects of the compounds on recombinant GCDH WT catalytic activity, a colorimetric assay based on the reduction of ferrocenium hexafluorophosphate (PcPF6) as an artificial electron acceptor was performed. Figure 6 depicts the stabilizing/activating effect of the final 25 compounds (10 μM) on GCDH WT. Notably, most compounds increased GCDH specific activity at a concentration of 10 μM. Compounds A29, B31, and B34 were the most effective, inducing activation of GCDH by increasing the specific activity over 41.2, 47.1, and 51.8%, respectively (*p* < 0.005, compared to no treatment). Multiple chemotypes displayed positive effects, with scaffold one being the most prevalent among the compounds that exhibited a statistically significant effect, representing approximately 60% (4 out of 7 compounds).

The orthogonal assays employed effectively validated the identified hits. Specifically, 88% (22/25) were confirmed using TSA with a Δ*T*m greater than 2 °C. Of the 25 compounds, 36% (9/25) exhibited significant effects on enzymatic activity, and 52% (13/25) displayed binding evidence by Trp quenching. Notably, compounds A49, A55, A71, B29, and B31 showed simultaneous effects on GCDH activity (exceeding a 25% change relative to untreated samples), protein stabilization (with Δ*T*_m_ score exceeding 3) and binding (as evidenced by ITC binding or a Trp quenching-derived *K*_d_ of less than 100 μM). These findings highlight them as key candidates for further drug development. Table 2 provides a summary of the results obtained for these five compounds, including their chemical structures. Importantly, no significant correlation was found between the compound’s solubility and its biochemical effects (Figure S2).

**Table 2:**
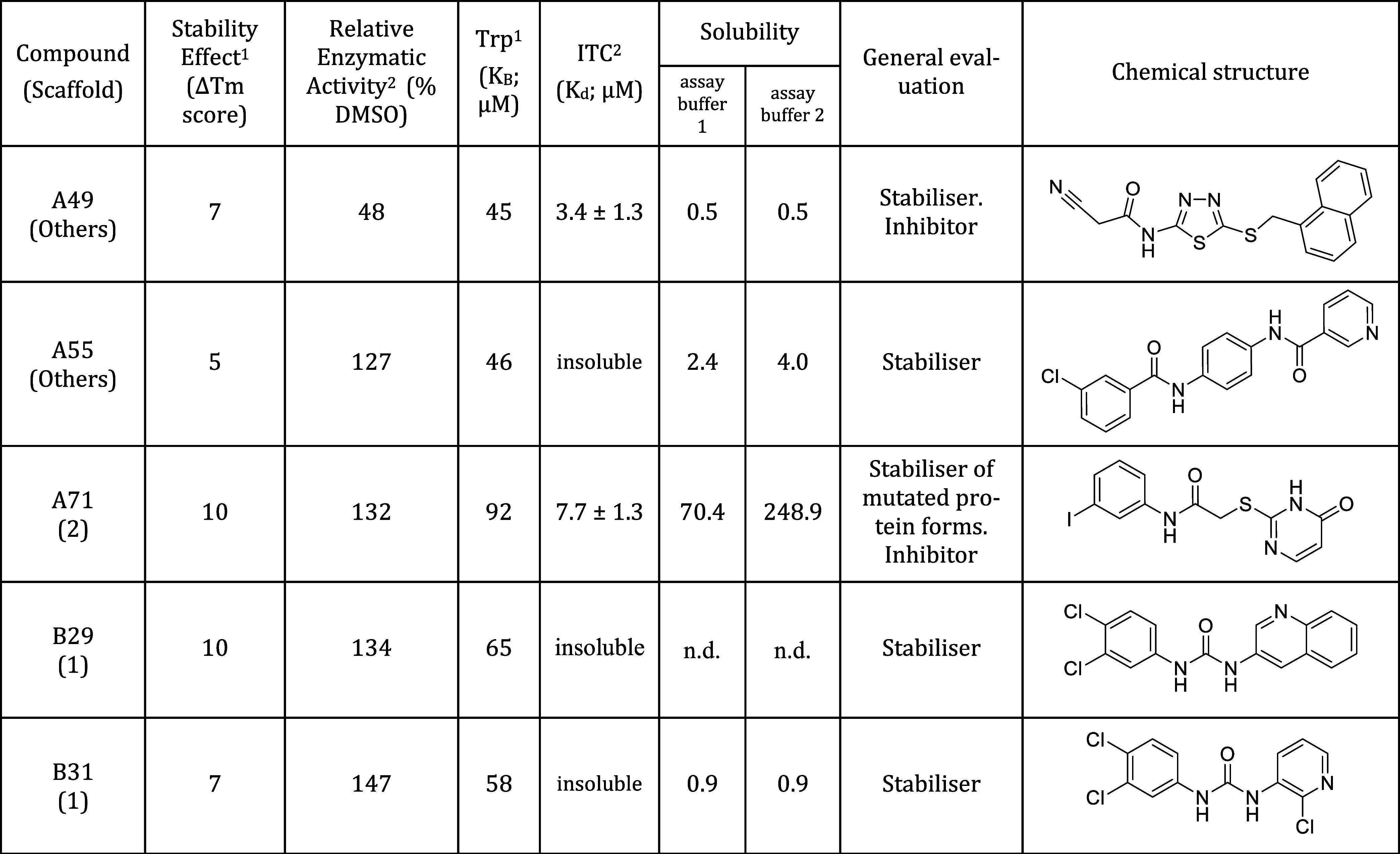
Summary of Biochemical and Biophysical Effects for the Final Five Lead Compounds, Including Chemical Structures^a^

a Scaffold, scaffold group; GCDH wild type (150 nM) activity was determined in the presence of 10 μM compound (in comparison to DMSO control); GCDH, glutaryl-CoA dehydrogenase; Stability effect, sum of Δ*T*_m_ scores for the different GCDH variant proteins tested; Δ*T*_m_ score, assigned based on the Δ*T*_m_ (change in melting temperature) for each protein variant, with scores of 1 given for Δ*T*_m_ > 1 °C, 2 for Δ*T*_m_ > 2 °C and 4 for Δ*T*_m_ > 3 °C; *K*_B_, binding affinity constant; *K*_d_, dissociation constant; Trp, tryptophan quenching; DSF, differential scanning fluorimetry; ITC, isothermal titration calorimetry; n.d., not detectable (no visible peak could be detected in the sample). Upperscript numbers 1 and 2 indicate the assay buffer used: assay buffer 1, 20 mM HEPES 200 mM NaCl (pH 7.0) and assay buffer 2, phosphate buffer (pH 7.2).

### Lead Validation and Effect on Disease Variants—Enzyme Kinetics

**Figure 7:**
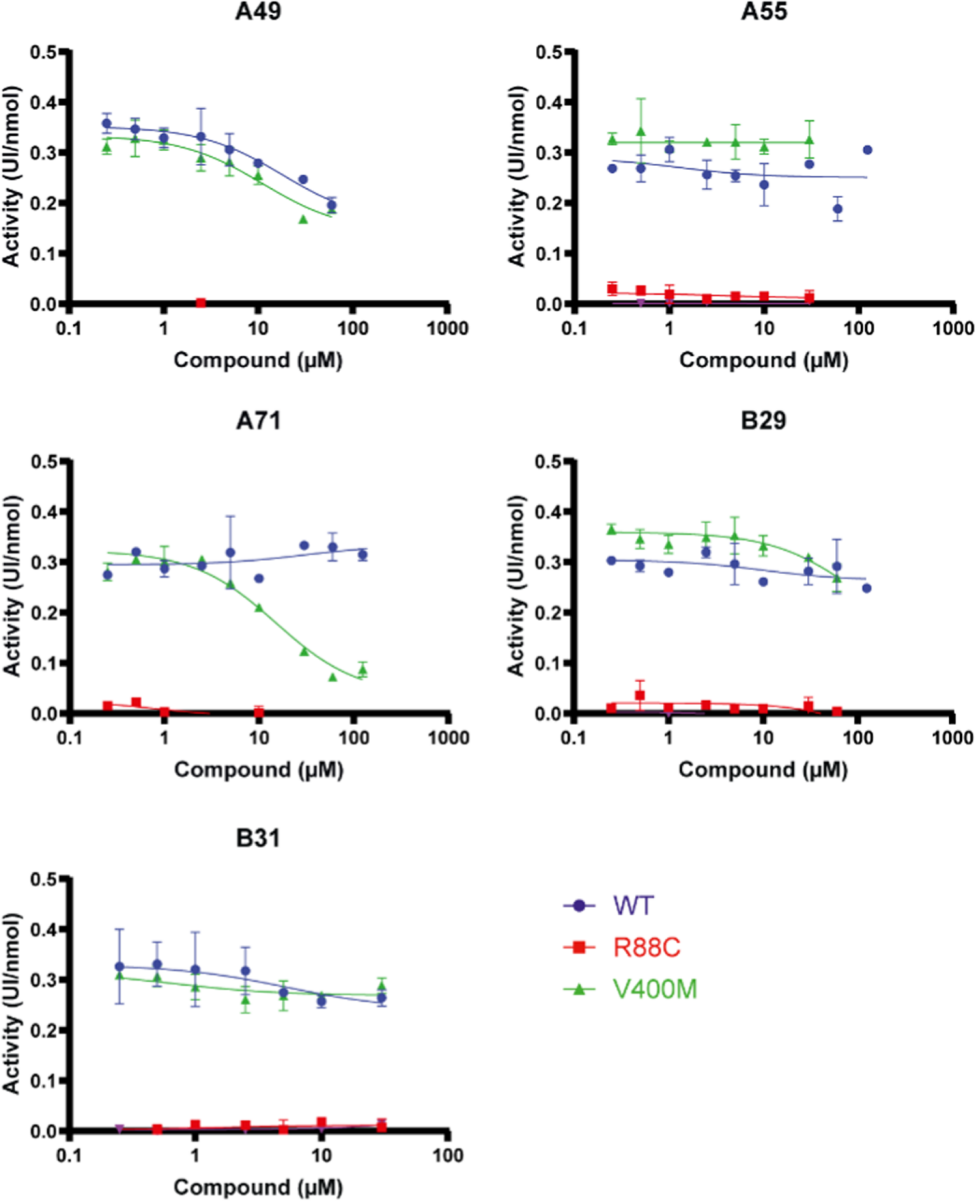
Effect of the 5 leading compounds on enzymatic activity of recombinant GCDH WT and variants R88C and V400M. Enzyme kinetics were measured in the presence of different compound concentrations (0.25–125 μM). Enzyme activity is shown as mean UI/nmol of protein (±standard deviation). Two experimental replicates were used. WT, wild type.

To further confirm the effect of the five lead compounds and determine their effects on disease variants, we performed a dose-dependent enzymatic assay using purified recombinant GCDH protein without a stabilizing MBP (maltose-binding protein)-tag (Figure 7).

No residual activity could be detected for variant A433E, and it was almost absent for variant R88C (Figure 7). Compounds A49 and B31 showed an inhibitory effect of GCDH WT, with a stronger inhibition for compound A49, which revealed an IC_50_ of 18 μM. V400M was inhibited by increasing concentrations of A49, A71, and B29, with the lowest IC_50_ corresponding to compound A71 (below 1 nM).

**Figure 8:**
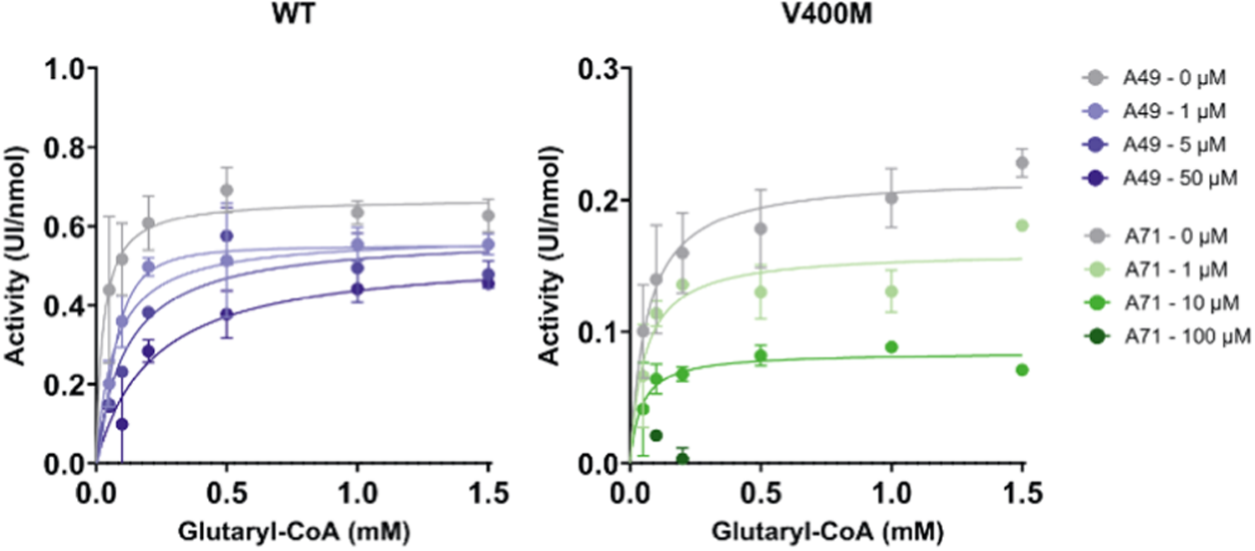
Competition assay for proteins GCDH WT with compound A49 (left, purple) and GCDH V400M and A71 (right, green). Enzyme kinetics were measured in the presence of different substrate concentrations (0–1.5 mM). Enzyme activity is shown as mean UI/nmol of protein (±standard deviation). A Michaelis–Menten equation was used to fit the data (line). Two experimental replicates were used. GCDH, glutaryl-CoA dehydrogenase; WT, wild type.

In order to shed light on the inhibitory mechanism and binding location behind the most promising molecules, we performed a competition assay at different compound concentrations and increasing concentrations of the substrate glutaryl-CoA. The assay was performed for the WT with compound A49 (concentrations 0, 0.5, 5, and 50 μM) and V400M with compound A71 (concentrations 0, 1, 10, and 100 μM), as shown in Figure 8.

The Michaelis–Menten kinetic analysis for the WT enzyme revealed that with increasing A49 compound concentrations there was a 10-fold increase in the Km (from 0.026 μM in the absence of the compound, to 0.22 μM in the presence of 50 μM) with a slight decrease in the Vmax (from 0.67 μM/s^–1^ in the absence of the compound, to 0.53 μM/s^–1^ in the presence of 50 μM). Variant V400M showed a clear noncompetitive interaction with A71, exhibiting no effect on the Km value (0.05 μM) and a lowering Vmax with increasing inhibitor concentrations, from 0.2 (no compound) to 0.08 μM/s^–1^ (10 μM A71). To validate the binding of A71 to V400M, ITC was performed and confirmed a binding interaction with a *K*_d_ of 117 ± 91 μM (Figure S3).

### Allosteric Site Binding—Thermal Shift Assay Using Allosteric Site Variants

**Figure 9:**
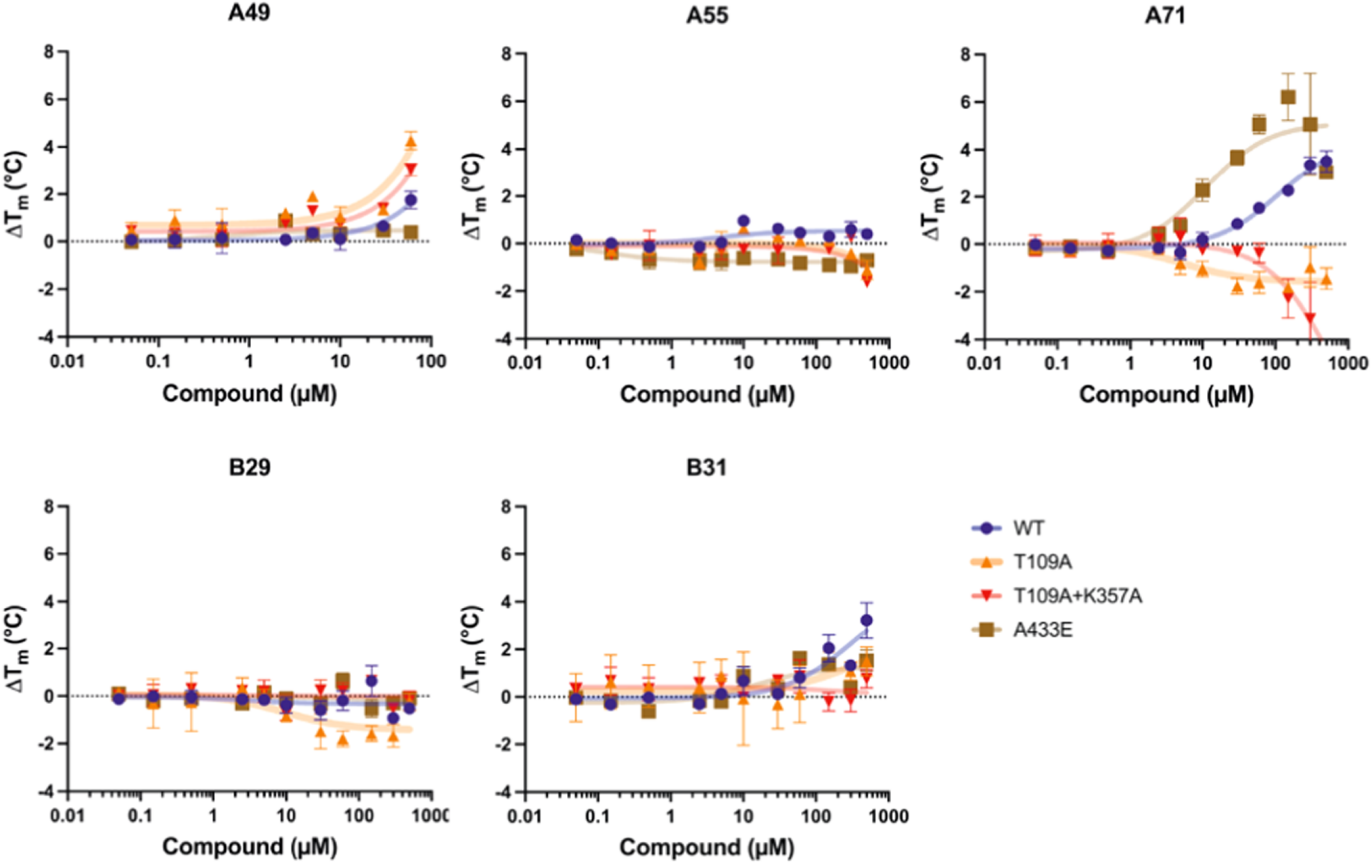
Dose-dependent effect on thermal stability of glutaryl-CoA dehydrogenase (GCDH) wild type (WT) and mutations mapping to the proposed allosteric binding site in the presence of lead compounds A49, A55, A71, B29, and B31. Thermal shift assays were performed for each protein variant (WT, A433E, and allosteric pocket mutant T109A, and double mutant T109A and K357A), using different compound concentrations from 0.05 to 500 μM. Variant A433E was used as an extra control for a pocket-independent mutation. A *T*_m_ (melting temperature: the temperature at which 50% of a protein is unfolded) was calculated for each concentration, and the thermal shifts (Δ*T*_m_s, difference of *T*_m_ in the presence and absence of compound) were plotted against compound concentration. Δ*T*ms are shown as mean and error bars corresponding to standard deviation (*n* = 3).

In order to verify compound binding at the allosteric site identified during docking, we generated specific pocket site variants. Specifically, we disrupted the polar contact involving the backbone nitrogen of Thr109 by substituting threonine with alanine (T109A). Additionally, we introduced a double mutant incorporating T109A and K357A to further disrupt potential hydrophobic interactions. The lead compounds were then tested in the newly generated mutants using TSA (Figures 9 and S5). Compounds A55 and B29 did not show significant thermal stability changes in the tested GCDH proteins (without MBP-tag). Compound A49 stabilized all tested protein variants, achieving the greatest stabilization (>4 °C) in the T109A single mutant at the highest compound concentration. Wild type and the pocket-independent variant A433E were used as controls. Compound A71 presented different effects depending on the mutation tested. Consistent with previous results, A71 stabilized both the WT and the disease variant A433E. However, this stabilizing effect was not observed with the pocket site mutations (T109A and T109A+K375A). Similarly, the stabilizing effect of compound B31 was absent in the double mutant GCDH T109A + K375A. These results support the existence of an interaction between the compounds A71 and B31 with the Thr109 and Lys357 residues, when bound to GCDH.

## Discussion and Conclusions

In this study, we used the proprietary SEE-Tx computational drug discovery platform to identify and exploit a previously uncharacterized putative allosteric site of the GCDH protein. Using a high-throughput in silico protocol, a virtual collection of about 3 million commercially available compounds was evaluated to identify and purchase virtual hits showing a high docking score that were tested for target validation. The subsequent in vitro hit rate based on binding and functional assays was remarkably high (22.9%). Compound solubility was not taken as a screening primary end point. However, considering the usual high number of false positives associated with small molecule aggregation,^32^ we attempted to assess the role of solubility within our experimental framework. We proceeded in a step-by-step screening cascade, initially incorporating two independent solubility predictions to increase our confidence in the selections made for the validation stages and later performed an experimental solubility assessment. We have excluded the role of aggregation and low solubility in hit selection, by performing a correlation analysis between the biochemical assays and solubility indicators (calculated log *S* and colloidal aggregation prediction; Figure S2).^32,33^ This approach led us to the identification of putative novel pharmacological chaperones, from which one potent hit exhibited an excellent binding potency (*K*_d_ = 3.4 μM) for GCDH. Besides this standout binder, four other compounds displayed concurrent effects on GCDH activity, potent protein stabilization, and strong binding. This combination of attributes marks them as lead candidates for continued drug advancement. Interestingly, and in agreement with the expectation for allosteric molecules, binding affinities do not correlate with functional activity. Allosteric modulators mostly exert their effects through conformational changes in target proteins, making the relationship between binding affinity and functional impact nonlinear and complex.^33,34^ While the majority seem to behave as silent binders (silent allosteric modulators; SAMs), others may activate the protein (positive allosteric modulators; PAMs) or inhibit the protein (negative allosteric modulators; NAMs). This underscores the value of exploring the effects of these compounds in more complex systems, such as cellular or animal models, to gather evidence on whether GCDH stabilization and function can be restored. Additionally, the effects observed when compound concentrations are above their solubility limits should be carefully considered. Particularly, the calculated ITC *K*_d_ for compound A49 (3.4 μM) is above its experimentally determined solubility (0.5 μM), which highlights the limitation of *K*_d_ determination. Alternative ITC protocols are necessary to validate the compound’s affinity.

To further validate the biological effect of the found hits in enzyme kinetics using an MBP-tag-free enzyme, we performed a dose-dependent enzymatic assay. The removal of the tag was important to avoid any of its influence on the protein stability and compound effects. We could observe variant-specific effects with the different compounds, which highlights the importance of understanding the misfolding mechanisms underlying each variant. With exception of A55, all compounds showed an inhibitory effect (Figure 7). In addition, a dose-dependent TSA assay was also performed with the untagged protein (Figure S4), which confirmed a dose-dependent stabilization effect for all compounds with the exception of A55. An enzyme kinetics competition assay, using GCDH substrate glutaryl-CoA and compound A71 on the previously responsive variant V400M, revealed a noncompetitive binding for the substrate pocket, supporting the identification of A71 as an allosteric regulator.^35,36^ To further substantiate the compound binding to the newly described allosteric pocket, mutations were introduced at the predicted hotspot interaction residues. Namely, T109A, and the double mutant T109A and K357A. The amino acid substitutions to alanine allow us to better assess each residue’s importance to compound binding, as alanine does not introduce additional functional groups or charge. The binding of A71 to the discovered allosteric site was further evidenced by the loss of induced stabilization upon mutation of the binding pocket residues, indicating the relevance of both residues for the compound interaction. It is important to note that although not desirable, the inhibitory effect of pharmacological chaperones is usually overcome by its stabilization effects. Intracellularly, increased stability often holds the protein available and retrieves an increase in its activity. Therefore, SAMs and NAMs are also important drug candidates. Typically, pharmacological chaperones act as reversible inhibitors that still allow for an equilibrium quantity of stable unbound enzyme available for substrate binding but they necessitate careful dosing regimens to maintain bioavailability of the active protein.^17^ Allosteric pharmacological chaperones that functionally behave as SAMs or PAMs offer, a priori, a wider therapeutic window because their stabilizing impact is not countered by activity loss.^17,18^ Our study to identify GA1 allosteric modulators for the treatment of GA1 builds on the success obtained with our computational platform to develop STAR small molecules that recover the activity β-Gal found to be impaired in GLB1-related disorders.^20,23^ It is worth noting that although low solubility was observed for four out of the five final lead compounds, we successfully reproduced biological effects during the validation stages in various assays, which supports the relevance of these hits. Nevertheless, compound A71, which exhibited the best solubility profile compared with the other four lead molecules, emerged as the most promising candidate, particularly as an allosteric binder. This fact underscores the importance of solubility in our evaluations, supporting the strategy of exploring analogous compounds with enhanced solubility in subsequent medicinal chemistry efforts.

Identifying and developing new pharmacological agents are notoriously time-consuming and expensive. High throughput screening (HTS) is the main discovery strategy employed by pharmaceutical companies. It is a powerful but costly technology that has delivered leads for 50% to 60% of the targets.^37^ However, the success rate could be even lower for unprecedented targets and HTS can be impractical in the absence of a reporter ligand or when binding does not necessarily imply a change in enzymatic activity, as is the case for allosteric pharmacological chaperones.^37,38^ The SEE-Tx platform is an efficient and cost-effective solution for allosteric drug discovery, offering broad applicability that we have exemplified for protein misfolding disorders. This strategy reveals hidden therapeutic opportunities, potentially bringing treatments to the market faster and, thus, improving patient outcomes.

Early diagnosis of GA1 by newborn screening is recommended for timely treatment to prevent irreversible neurological impairment.^3^ Timely metabolic treatment involves a low-lysine diet until the age of 6 years with a lysine-free, tryptophan-reduced, arginine-containing amino acid mixture plus carnitine supplementation.^3^ Maintenance therapy thus aims to protect the striatum of GCDH deficient patients during early development by reducing cerebral lysine influx and clearing glutaryl-CoA from brain cell mitochondria.^4^ Additional intensified emergency treatment during an acute encephalopathic crisis is recommended to stabilize young brain tissue during intervals of physiologic stress.^3,39^ A meta-analysis by Boy et al.^1^ involving 647 patients with GA1 demonstrated the positive effect of newborn screening combined with early metabolic maintenance treatment and transient emergency treatment on neurological motor outcomes.^3^ However, some cases are progressive despite appropriate treatment.^3^ Furthermore, Märtner et al. recently showed that the biochemical phenotype of individuals with GA1 affects cognitive impairment.^5^ Biochemical high excreter subtype patients were at higher risk of cognitive impairment and had poorer prognoses than patients with the low excreter clinical phenotype, despite newborn screening and regardless of the quality of treatment and striatal damage.^5^ Therefore, more effective and novel treatment options are needed.

Our results identified potential hit compounds that could enter further steps of the drug development process for a new GA1 therapy. We have identified three distinct groups based on structural clustering. Scaffolds 1 and 2 are fairly homogeneous in structure, while the remaining compounds (“others”), though structurally diverse, share some similarities and could lead to several distinct chemical series. Specifically, Scaffold 1 contains several examples from which SAR (Structure–Activity Relationship) can be inferred based on the screening outcomes (Table S7), aligning well with the predicted binding mode at the new allosteric site (Tables S7–S9). A comparison of the scaffold 1-derived compounds to their parent compounds, highlighting changes that led to stability loss, is shown in Table S8. Additionally, we established a detailed comparison between the predicted binding modes of two representative compounds, emphasizing the significance of specific structural features for effective allosteric modulation (Table S9). For scaffold 2, which contains promising compounds, further exploration and testing of analogues are important for the development of SAR and series validation. The remaining compounds, which could not be clustered, are chemically diverse but offer valuable insights, as all ligands exploit the same binding site, allowing for knowledge transfer among clusters.

The excellent stabilizing effects, the high hit rate (>20%), the active compounds’ chemical diversity, and the structure–activity relationships derived from the primary screening offer excellent perspectives for the hit-to-lead and lead optimization stages. Experimental solubility assays revealed that most of the discovered lead compounds present low solubility (Tables 2 and S10). As mentioned, this factor should be taken into consideration for data interpretation, as solubility can affect the assay readouts. The compounds’ solubility profiles should also be the basis for future medicinal chemistry approaches during lead optimization stages. By focusing on allosteric site regulators, we hope to mitigate the risk of cross-reactivity. To fully leverage the computational drug discovery platform in this context, it is crucial to conduct additional research to validate the binding interactions, further elucidate the mechanism of action, selectivity, and cross-reactivity assays, and optimize the binding affinities and ADMET (Administration, Distribution, Metabolism, Excretion and Toxicity) properties. Binding to the predicted allosteric pocket of GCDH could be experimentally corroborated by using X-ray crystallography or cryogenic electron microscopy techniques. Nevertheless, both approaches used for allosteric pocket validation (enzyme substrate competition assay and compound stability upon pocket mutation) provided further support for the SEE-Tx identified allosteric binding site. Moreover, while the identified compounds demonstrated promising binding and functional activity, these may benefit from follow-up medicinal chemistry approaches, and additional investigations are required to confirm their dose-dependent effects and drug-like properties, including pharmacokinetic and toxicological assessments. This study underscores the potential of the SEE-Tx® computational strategy in identifying allosteric regulators acting as pharmacological chaperones, offering a effective approach to addressing protein misfolding disorders and advancing therapeutic discovery.

## Experimental Section

### Identification of STARs

The innovative proprietary drug discovery platform SEE-Tx was applied to identify druggable binding sites different from those of the active site. The structure of human glutaryl-CoA dehydrogenase in complex with the cofactor flavin adenine dinucleotide (FAD; protein databank [PDB] code1SIQ)^30^ was used as the starting point for the simulations, ensuring the generation of the biological unit (homotetramer: dimer of dimers) from the single chain found in the asymmetric unit. FAD was kept in the MDmix simulation because the aim was to find allosteric ligands that would not compete with the substrate or the cofactor. The druggability of the putative allosteric binding site identified was confirmed with a physics-based method consisting of molecular dynamics simulations of the protein in organic-aqueous solvent mixtures.^24,29,40^ The same method was used to identify key interactions (binding hot spots), which were used as pharmacophoric restraints to guide molecular docking-based virtual screening with the *rDock* program.^25,28,29^ A virtual collection of 2,705,434 nonredundant chemical compounds commercially available from 6 vendors: Asinex (Russia), Enamine (Ukraine), Life Chemicals (Ukraine), Princeton Biomolecular Research Inc. (USA), Specs (Netherlands), Vitas-M Laboratory (Russia) along with compounds from our library (GAIN DB) was evaluated using the standard scoring function, pharmacophoric restraints and a high-throughput protocol.^41^ This resulted in 2,200 molecules that fulfilled the four mandatory pharmacophoric restraints and obtained an intermolecular docking score lower than −18.0 units, indicating a strong affinity between the docked molecules and the target site. These compounds were ranked by normalized score (intermolecular docking score divided by the number of nonhydrogen atoms) and clustered by similarity based on MACCS fingerprints. Visual inspection of the resulting set of diverse and top-ranked compounds led to the selection and purchase of 94 compounds. The visual inspection step was used to correct deficiencies in the rDock scoring function, particularly in the SCORE.INTRA term (removing docking poses with conformational strain) and insufficiently penalized desolvation cost (removing ligands that bury polar groups in hydrophobic environments). The selected compounds were screened using thermal stability assays^42^ and further validated by purchasing and testing hit analogues to obtain structure–activity relationships (SAR). All screening compounds are >90% pure, as confirmed by LC-MS and/or H NMR analysis (Tables S1 and S2). All lead compounds are >95% pure by HPLC analysis (Table S2 and Figure S1).

### Protein Expression and Purification

The cDNA of human GCDH (clone ID HsCD00372664 from the Plasmid Information Database [PlasmID Repository, Boston]) was cloned into the prokaryotic pMAL-c2X expression vector (New England Biolabs), encoding an N-terminal maltose-binding protein (MBP).^43^ The coding sequence for the first 44 protein residues was removed from the pMAL-c2X insert, as these correspond to the mitochondrial-targeting sequence amino acids and are not required for protein expression. GCDH mutants (R88C, V400M, E414 K, A433E) were generated using the QuikChange site-directed mutagenesis kit (Stratagene).^44^ Authenticity of mutagenesis was verified by DNA sequencing. The *E. coli* strain BL21 DE3 was transformed with expression plasmids for wild type and variant GCDH. Bacteria were grown to mid-exponential phase at 20 °C in Lysogeny broth (LB) medium supplemented with 100 μg/mL ampicillin, 5 mL/L glucose (v/v 20%) and 2.5 g/L K_2_HPO_4_. Overexpression of wild type and variant GCDH-MBP fusion proteins was induced with 0.3 mM isopropylthio-β-d-galactoside (IPTG) with the addition of 1 μg/mL Riboflavin (Sigma) at 28 °C. All protein tetramers were purified from cell lysates by MBPTrap affinity chromatography (GE Healthcare), using column buffer (20 mM Tris-HCl, pH 7.4, 200 mM NaCl, 1 mM EDTA, 1 mM DTT) and elution buffer (column buffer supplemented with 10 mM maltose), followed by size-exclusion chromatography (SEC) using a HiLoad 16/600 Superdex 200 column (GE Healthcare) and a 20 mM HEPES buffer containing 200 mM NaCl (pH 7.0).^45^ In order to increase the stability and reproducibility of the GCDH protein, the MBP-tag was initially not cleaved. Alternatively, for lead validation studies, the protein was cut with Factor Xa for variant A433E or cloned into a pET-28a vector for expression with a C-terminal His6-tag. Site-directed mutagenesis was performed to replace the first 2 GCDH residues by Met-Ala-Asp to ensure purification of His6-C-terminally tagged variants. The first coding residues can be determinant for GCDH protein stability.^8^ His6-tagged proteins were purified following an equivalent 2-step purification approach, where a HisTrap affinity column (Cytiva) was used instead of MBPTrap, followed by SEC. A 50 mM potassium phosphate buffer (pH 7.2) was used for SEC of His6-tagged proteins.

### Thermal Shift Assay

Thermal shift assays (TSA) were performed as differential scanning fluorimetry (DSF) measurements using a high-throughput approach with a 7900 HT Fast Real-Time PCR System (Applied Biosystems) equipped with Micro AmpTM optical 96-well reaction plates or a QuantStudio 12K Flex System (Thermo Fisher), equipped with a 384-well block. The heat-induced denaturation was performed using purified protein (MBP-tagged 0.6 mg/mL; untagged/His6-tag 0.2 mg/mL) in the purification buffer and Sypro Orange (Invitrogen, dilution 1:1000) in the temperature range from 25 to 60 °C (MBP-tagged protein) or 30 to 70 °C (untagged/His6-tagged protein) at a heating rate of 1 °C/min. The influence of the compounds on the thermal stability of GCDH wild type and mutants was analyzed at different concentrations. For each sample, three to six replicates were analyzed. Transition midpoints (Δ*T*m) were determined after applying a nonlinear regression model using GraphPad Prism. Variant E414 K presented a biphasic TSA profile, and a biphasic model was used for the calculation of the melting temperatures. Due to the different analysis required, this variant was excluded after the first screening series. Statistical analysis was performed using one-way ANOVA followed by a Dunnett post-test (GraphPad Prism 9.0).

### Tryptophan Quenching

UV–visible spectra (295–450 nm) were obtained for purified GCDH wild type protein (5 μM) in the presence of the different compounds by using a Cary Eclipse fluorescence spectrophotometer (Varian). A total of 16 different compound concentrations (0, 1, 3, 5, 8, 10, 15, 20, 25, 30, 35, 40, 45, 50, 75, and 100 μM) were titrated into the protein as different spectra. Tryptophan fluorescence at 350 nM was selected for further evaluation. Linear regressions were calculated for tryptophan fluorescence over the different compound concentrations using GraphPad Prism and the slope inverse was used as a reference for the compounds’ binding affinity (*K*_B_).

### Isothermal Titration Calorimetry

All titrations were performed using a MicroCal PEAQ-ITC (Malvern) at 20 °C. Measurements were done with titrant concentrations at 500 μM for all compounds. Cell samples contained purified recombinant WT GCDH, or GCDH V400M, at 30 μM. The runs consisted of 19 injections with a volume of 2 μL and a duration of 4 s each. The raw data were processed and integrated using MicroCal PEAQ-ITC Analysis Software v.1.0.0.1259 (Malvern). The resulting fitting values from Δ*H*/mol plotted over the molar ratio were exported, and a graphical representation was prepared using GraphPad Prism 9.

### GCDH Colorimetric Activity Assay

Recombinant GCDH protein activity was determined following reduction of the artificial electron acceptor (ferrocenium hexafluorophosphate, Sigma) at 300 nm. The enzymatic reaction was tested at room temperature using a reaction mixture containing 200 μM ferrocenium, 100 μM Glutaryl-CoA, and 100 μM FAD in 50 mM potassium phosphate buffer (pH 7.2). The reaction was started by adding the purified protein (150–180 nM final concentration), and the mixture was monitored for 5 min using a CLARIOstar spectrophotometer (BMG Labtech). Alternatively, the assay was started with the addition of glutary-coA during the enzyme kinetic validation studies. Statistical analysis was performed by applying 1-way ANOVA followed by Dunnett’s multiple comparison test using GraphPad Prism 9. Data are shown as mean ± standard deviation.

### Apparent Solubility Assay

Compound stock DMSO solutions (10 mM) were used to determine the apparent solubility of compounds in two independent buffers: 20 mM HEPES buffer containing 200 mM NaCl (pH 7.0) and PBS (pH 7.2). The control standard (Std) compound was diclofenac sodium (Sigma-Aldrich) in DMSO at a concentration of 10 mM.

The assay was performed in duplicate, and samples were placed into a plate shaker at 25 °C and 1100 rpm for 2 h. After 2 h, the samples were filtered using the Vacuum Manifold ORVMN96 (Orochem). After filtration, an aliquot of each sample was tested by LC-MS/MS in the presence of a mixture of H_2_O and acetonitrile (1:1 in v/v) with 1% DMSO.^46^

The filtrate was analyzed and quantified using the ratio of the peak area of the compound to the known concentration of that compound. Solubility values of the samples and control compound were calculated as follows:
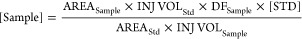
where AREA refers to the peak area, INJ VOL is the respective injection volume for the standard or sample, and DF is the dilution factor. The DF was adjusted according to the solubility value and the LC-MS signal response.

## Supporting Information Available

List of the 170 compounds including the respective vendor, vendor identifier, and purity (Table S1); purity data of lead compounds (Table S2); Library A Screen Thermal Shift Assay (94 compounds) (Table S3); Library B Screen Thermal Shift Assay (76 compounds) (Table S4); chemical properties of the final 25-compound library (Table S5); ITC binding and thermodynamics parameters with binding compounds (Table S6); SAR data of commercially available compounds with variations of scaffold 1 (Table S7); SAR data of compounds available with variations of scaffold 1 compared to the corresponding parent compound selected as a screening hit (Table S8); predicted binding mode of two representative compounds from scaffold 1 in the allosteric pocket (Table S9); solubility data of lead compounds (Table S10); HPLC traces for lead compounds (Figure S1); absence of significant correlation between compounds’ solubility and biochemical data (Figure S2); isothermal titration calorimetry (ITC) analysis of the interaction of glutaryl-CoA dehydrogenase (GCDH) variant V400M with compound A71 (Figure S3); dose-dependent effect on thermal stability of glutaryl-CoA dehydrogenase (GCDH) wild type (WT) and variants in the presence of compounds A49, A55, A71, B29, and B31 (Figure S4); and dose-dependent effect on thermal stability of glutaryl-CoA dehydrogenase (GCDH) wild type (WT) and mutations mapping to the proposed allosteric binding site in the presence of lead compounds A49, A55, A71, B29, and B31 (Figure S5) (PDF)
Molecular formula strings (SMILES) (CSV)

## Abbreviations Used

DSF: differential scanning fluorimetry
GA1: glutaric acidemia type 1
GCase: glucocerebrosidase
GCDH: glutaryl-CoA dehydrogenase
ITC: isothermal titration calorimetry
*K*_d_: dissociation constant
PCT: pharmacological chaperone therapy
SEE-Tx: site-directed enzyme enhancement therapy
STAR: structure-targeted allosteric regulator
