# Supplementary material for: Use of the Novel Site-Directed Enzyme Enhancement Therapy (SEE-Tx) Drug Discovery Platform to Identify Pharmacological Chaperones for Glutaric Acidemia Type 1
Source: J Med Chem. 2024 Sep 23;67(19):17087–100. doi: 10.1021/acs.jmedchem.4c00292 (PMC11472340; doi:10.1021/acs.jmedchem.4c00292)

Supporting Information

Use of the Novel Site-Directed Enzyme Enhancement Therapy (SEE-Tx®) Drug Discovery Platform to Identify Pharmacological Chaperones for Glutaric Acidemia Type 1

Madalena Barroso,[¥,#] Alexandra Puchwein-Schwepcke,[†,&,#], Lars Buettner,[§] Ingrid Goebel,[¥] Katrin Küchler,[¥] Ania C. Muntau,[‖,¢] Aida Delgado,[‡] Ana M. Garcia-Collazo,[‡] Marc Martinell,[⊥] Xavier Barril,[‡] Elena Cubero[‡,*] and Søren W. Gersting[¥,¢,*]

[¥]University Children's Research, UCR@Kinder-UKE, University Medical Center Hamburg-Eppendorf, Hamburg 20246, Germany
[†]Department of Molecular Pediatrics, Dr. von Hauner Children's Hospital, Ludwig-Maximilians-University, Munich 80337, Germany
[&] Department of Pediatric Neurology and Developmental Medicine, University Children's Hospital Basel UKBB, Basel 4031, Switzerland
[§] Pharmaceutical Development Biologicals, Boehringer Ingelheim Pharma GmbH & Co. KG, Biberach an der Riss 88397, Germany
[‖]University Children's Hospital, University Medical Center Hamburg-Eppendorf, Hamburg 20246, Germany
[¢]German Center for Child and Adolescent Health (DZKJ), partner site Hamburg, University Medical Center Hamburg-Eppendorf, Hamburg 20246, Germany
[‡]Gain Therapeutics Sucursal en España, Parc Científic de Barcelona, Barcelona 08028, Spain
[⊥]Minoryx Therapeutics S.L. TecnoCampus Mataró-Maresme, Mataró, Barcelona 08302, Spain

[#]*should be considered joint First Authors*
[*]*should be considered joint Corresponding Authors*

Corresponding Author

E. Cubero Email: ecubero@gaintherapeutics.com, Tel: +34 93 403 55 27.

S.W. Gersting Email: gersting@uke.de, Tel: +49 40 741055330.

Contents

Table S1. List of the 170 compounds (sorted by vendor).

Table S2. Purity data of lead compounds.

Table S3. Library A Screen Thermal Shift Assay (94 compounds).

Table S4. Library B Screen Thermal Shift Assay (76 compounds).

Table S5. Chemical properties of the final 25-compound library.

Table S6. ITC binding and thermodynamics parameters with binding compounds.

Table S7. SAR data of commercially available compounds with variations of scaffold 1.

Table S8. SAR data of compounds available with variations of scaffold 1 compared to the corresponding parent compound selected as a screening hit.

Table S9. Predicted binding mode of two representative compounds from scaffold 1 in the allosteric pocket.

Table S10. Solubility data of lead compounds.

Figure S1. HPLC traces for lead compounds.

Figure S2. Absence of significant correlation between compounds' solubility and biochemical data.

Figure S3. Isothermal titration calorimetry (ITC) analysis of the interaction of glutaryl-CoA dehydrogenase (GCDH) variant V400M with compound A71.

Figure S4. Dose-dependent effect on thermal stability of glutaryl-CoA dehydrogenase (GCDH) wild type (WT) and variants in the presence of compounds A49, A55, A71, B29, and B31.

Figure S5. Dose-dependent effect on thermal stability of glutaryl-CoA dehydrogenase (GCDH) wild type (WT) and mutations mapping to the proposed allosteric binding site in the presence of lead compounds A49, A55, A71, B29, and B31.

Table S1. List of the 170 compounds (sorted by vendor). List of 170 compounds including the respective vendor, vendor identifier, and purity. All stock screening compounds are confirmed to be over 90% pure, as determined by LC-MS or H-NMR analysis by the respective vendor.

Compound	Vendor	Vendor ID	Purity
A76	Asinex	BAS 00665338	>90%
A77	Asinex	ASN 04889328	>90%
A78	Asinex	BAS 05881565	>90%
A79	Asinex	BAS 06739946	>90%
A80	Asinex	BAS 06838180	>90%
A81	Asinex	BAS 10846657	>90%
A82	Asinex	BAS 06739813	>90%
A83	Asinex	SYN 19995351	>90%
A84	Asinex	BAS 04360735	>90%
A85	Asinex	SYN 20059486	>90%
A86	Asinex	SYN 20004362	>90%
A87	Asinex	BAS 00790330	>90%
A88	Asinex	ASN 07439067	>90%
A89	Asinex	BAS 05346789	>90%
A90	Asinex	ASN 15349018	>90%
A91	Asinex	BAS 00829293	>90%
A92	Asinex	BAS 03034503	>90%
A93	Asinex	BAS 03819322	>90%
A94	Asinex	ASN 05116676	>90%
A14	Enamine	T5303036	>90%
A15	Enamine	T5776133	>90%
A16	Enamine	T5991214	>90%
A17	Enamine	T6164224	>90%
A18	Enamine	T6510138	>90%
A19	Enamine	Z44592326	>90%
A20	Enamine	Z27485993	>90%
A21	Enamine	Z45798280	>90%
A22	Enamine	T6473045	>90%
A23	Enamine	T6539405	>90%
A24	Enamine	T6637486	>90%
A25	Enamine	T6818629	>90%
A26	Enamine	T6898791	>90%
A27	Enamine	T6839056	>90%
A28	Enamine	T6426053	>90%
B1	Enamine	Z18410429	>90%
B10	Enamine	PB26497610	>90%
B11	Enamine	Z85526788	>90%
B12	Enamine	PB31868576	>90%
B13	Enamine	PB194940668	>90%
B14	Enamine	Z994284856	>90%
B15	Enamine	Z994268484	>90%

Compound	Vendor	Vendor ID	Purity
B16	Enamine	Z1229754461	>90%
B17	Enamine	BBV-32862719	>90%
B18	Enamine	BBV-34579992	>90%
B2	Enamine	PB57067052	>90%
B3	Enamine	Z44591822	>90%
B4	Enamine	Z18413201	>90%
B40	Enamine	T5237652	>90%
B41	Enamine	T5888485	>90%
B42	Enamine	T5252217	>90%
B43	Enamine	T5534720	>90%
B44	Enamine	T5767856	>90%
B45	Enamine	T6093647	>90%
B49	Enamine	T5832161	>90%
B5	Enamine	Z30624178	>90%
B52	Enamine	Z1303462797	>90%
B58	Enamine	BBV-34328724	>90%
B59	Enamine	PB71658583	>90%
B6	Enamine	Z53791615	>90%
B60	Enamine	PB44386616	>90%
B61	Enamine	BBV-32484543	>90%
B62	Enamine	BBV-24869342	>90%
B63	Enamine	BBV-32094146	>90%
B64	Enamine	BBV-34660354	>90%
B65	Enamine	Z994975086	>90%
B66	Enamine	BBV-074111	>90%
B67	Enamine	PB28218271	>90%
B68	Enamine	PB68300808	>90%
B69	Enamine	PB653671118	>90%
B7	Enamine	BBV-39251086	>90%
B70	Enamine	BBV-40593193	>90%
B71	Enamine	Z913258822	>90%
B72	Enamine	PB27870413	>90%
B73	Enamine	BBV-39188579	>90%
B74	Enamine	PB30250385	>90%
B75	Enamine	Z296581050	>90%
B76	Enamine	BBV-24908749	>90%
B8	Enamine	Z358323038	>90%
B9	Enamine	Z425748620	>90%
A29	LifeChemicals	F0227-0373	>90%
A30	LifeChemicals	F2024-0147	>90%
A31	LifeChemicals	F2024-1709	>90%
A32	LifeChemicals	F2024-1969	>90%
A33	LifeChemicals	F2285-0130	>90%
A34	LifeChemicals	F2700-0431	>90%
A35	LifeChemicals	F2700-0463	>90%

Compound	Vendor	Vendor ID	Purity
A36	LifeChemicals	F2749-0135	>90%
A37	LifeChemicals	F3146-0231	>90%
A38	LifeChemicals	F3217-0114	>90%
A39	LifeChemicals	F5382-0001	>90%
A40	LifeChemicals	F5382-0816	>90%
A41	LifeChemicals	F5458-0077	>90%
A42	LifeChemicals	F5458-0079	>90%
A43	LifeChemicals	F5791-3559	>90%
A44	LifeChemicals	F5847-0232	>90%
A45	LifeChemicals	F5857-1162	>90%
A46	LifeChemicals	F5857-1484	>90%
A47	LifeChemicals	F5965-0044	>90%
A48	LifeChemicals	F6089-7876	>90%
B53	Gain DB	INN207F	>90%
B54	Gain DB	INN215F	>90%
B55	Gain DB	INN246F	>90%
B56	Gain DB	INN275sub	>90%
B57	Gain DB	MT-15	>90%
A49	PrincetonBio	OSSK_469186	>90%
A50	PrincetonBio	OSSK_498338	>90%
A51	PrincetonBio	OSSK_721441	>90%
A52	PrincetonBio	OSSL_094177	>90%
A53	PrincetonBio	OSSL_093713	>90%
A54	PrincetonBio	OSSK_970104	>90%
A55	PrincetonBio	OSSK_315673	>90%
A56	PrincetonBio	OSSK_734443	>90%
A57	PrincetonBio	OSSL_313388	>90%
A58	PrincetonBio	OSSK_580719	>90%
A59	PrincetonBio	OSSK_336666	>90%
A60	PrincetonBio	OSSK_796998	>90%
A61	PrincetonBio	OSSK_342618	>90%
A62	PrincetonBio	OSSK_367243	>90%
A63	PrincetonBio	OSSK_678075	>90%
A64	PrincetonBio	OSSK_337992	>90%
A65	PrincetonBio	OSSK_679249	>90%
A66	PrincetonBio	OSSL_145079	>90%
A67	PrincetonBio	OSSL_234617	>90%
A68	PrincetonBio	OSSK_359857	>90%
A69	PrincetonBio	OSSL_241119	>90%
A70	PrincetonBio	OSSK_314926	>90%
A71	PrincetonBio	OSSL_031877	>90%
A72	PrincetonBio	OSSL_022632	>90%
B19	PrincetonBio	OSSK_480204	>90%
B20	PrincetonBio	OSSK_480208	>90%
B21	PrincetonBio	OSSK_186256	>90%

Compound	Vendor	Vendor ID	Purity
B22	PrincetonBio	OSSK_136446	>90%
B23	PrincetonBio	OSSK_214382	>90%
B24	PrincetonBio	OSSL_302105	>90%
B25	PrincetonBio	OSSK_378152	>90%
B26	PrincetonBio	OSSL_627106	>90%
B27	PrincetonBio	OSSK_731458	>90%
B28	PrincetonBio	OSSL_229581	>90%
B29	PrincetonBio	OSSK_674119	>90%
B30	PrincetonBio	OSSL_051904	>90%
B31	PrincetonBio	OSSL_204846	>90%
B32	PrincetonBio	OSSK_366057	>90%
B33	PrincetonBio	OSSL_047978	>90%
B34	PrincetonBio	OSSK_361387	>90%
B35	PrincetonBio	OSSL_239714	>90%
B36	PrincetonBio	OSSK_604850	>90%
B37	PrincetonBio	OSSL_052464	>90%
B38	PrincetonBio	OSSK_332248	>90%
B39	PrincetonBio	OSSL_145418	>90%
A1	Specs	AG-205/12229327	>90%
A10	Specs	AH-487/42143753	>90%
A11	Specs	AH-034/11366237	>90%
A12	Specs	AH-487/42923082	>90%
A13	Specs	AK-918/42486633	>90%
A2	Specs	AJ-292/41694744	>90%
A3	Specs	AH-487/42142867	>90%
A4	Specs	AK-918/41945450	>90%
A5	Specs	AH-487/42145416	>90%
A6	Specs	AG-690/40082109	>90%
A7	Specs	AN-465/42766923	>90%
A73	Specs	AN-465/42834501	>90%
A74	Specs	AP-853/42939130	>90%
A75	Specs	AJ-292/41685768	>90%
A8	Specs	AN-465/41587303	>90%
A9	Specs	AH-487/42194530	>90%
B46	Specs	AJ-292/14921023	>90%
B47	Specs	AK-968/40162671	>90%
B48	Specs	AN-465/42833690	>90%
B50	Specs	AG-205/40094633	>90%
B51	Vitas-M Lab	STK234215	>90%

Purity has been determined by LC-MS and/or ^{1}H-NMR (data available upon request).

Table S2. Purity data of lead compounds. All lead compounds exhibit a purity greater than 95%, as determined by HPLC-MS analysis performed during this work. Purity is indicated as % of peak area, obtained by integration of HPLC traces (Figure S1). The signal with the highest area was subjected to mass spectrometry to verify the molecular weight, ensuring it corresponds to the expected compound. The measured masses are then compared to their respective calculated values.

Compound	Purity (%)	Mass found	Mass calculated
A49	98.71	339.2 [M-H]-	340.05 (m/z)
A55	99.11	351.9 [M+H]+	351.08 (m/z)
A71	97.35	386.3 [M+H]-	387.20 (m/z)
B29	99.10	332.0 [M+H]+	331.03 (m/z)
B31	95.80	314.2 [M+H]-	314.97 (m/z)

Purity has been determined by HPLC-MS assays.

Table S3. Library A Screen Thermal Shift Assay (94 compounds). The thermal shift observed between the assay melting points of GCDH wild-type and four GCDH variants (R88C, V400M, E414K and A433E) in the presence and absence of compound (ΔTm) is shown for each compound at 3 different concentrations. Graphical representations on the rightmost column depict the transition of the ΔTm for the 3 concentrations in ascending order (minimum [0°C] and maximum [3°C] ΔTm values were set).

GCDH WT

	ΔTm (°C)			
Compound	10 µM	30 µM	100 µM	
1	0.54	0.51	0.49	
2	0.50	0.66	0.62	
3	1.10	0.17	-0.73	
4	0.89	0.99	0.87	
5	0.89	1.81	-0.39	
6	0.83	0.94	1.12	
7	0.83	0.94	1.12	
8	0.30	0.32	0.26	
9	0.59	0.49	0.61	
10	0.73	1.49	-2.42	
11	1.02	0.81	0.38	
12	1.29	1.10	1.25	
13	1.05	1.06	0.50	
14	1.01	0.99	0.70	
15	0.81	0.79	0.32	
16	0.53	0.62	0.33	
17	0.47	0.51	-0.14	
18	0.75	0.60	-0.18	
19	1.31	1.86	3.82	
20	1.36	1.64	0.96	
21	0.94	1.07	1.13	
22	0.83	0.68	0.44	
23	0.68	0.63	0.98	
24	0.59	0.35	0.58	
25	1.82	2.45	2.41	
26	0.80	0.73	1.07	
27	1.18	0.65	1.16	
28	0.82	0.39	0.81	
29	1.29	1.81	1.95	
30	0.00	-0.07	1.15	
31	0.66	0.63	0.98	
32	0.34	0.43	0.73	
33	0.50	0.72	1.90	
34	1.37	3.30	4.01	
35	2.53	3.01	2.56	
36	0.46	0.30	0.43	
37	0.18	0.52	0.65	
38	0.81	0.92	1.01	
39	0.44	0.29	0.31	
40	0.57	0.29	0.25	
41	1.05	2.39	4.37	
42	0.63	0.84	0.89	
43	0.72	0.89	0.54	
44	0.44	0.77	0.36	
45	-0.94	-0.76	-0.71	
46	-1.04	-0.58	-0.05	
47	-0.57	0.45	0.85	
48	-1.19	-0.85	-0.85	
49	-0.11	0.08	0.76	

	ΔTm (°C)			
Compound	10 µM	30 µM	100 µM	
50	-0.44	-0.51	n.d.	
51	0.90	1.09	0.03	
52	-0.51	0.56	-0.13	
53	-0.56	-0.84	-1.06	
54	0.40	0.71	-0.01	
55	3.04	1.92	1.99	
56	-0.94	-0.77	-1.11	
57	0.50	1.08	1.22	
58	-0.11	0.01	-1.60	
59	0.59	-0.07	2.20	
60	2.38	-1.17	3.15	
61	-0.27	-0.22	-0.12	
62	0.85	-0.49	-0.07	
63	0.59	-0.16	0.45	
64	-0.64	-1.06	-1.88	
65	-0.18	-0.60	-0.79	
66	0.31	0.18	0.04	
67	0.34	0.07	0.37	
68	-0.37	-0.01	-0.03	
69	-0.02	-0.12	-0.08	
70	0.00	0.14	0.75	
71	0.56	0.79	1.05	
72	-0.40	-0.45	-0.63	
73	-0.55	-0.71	-1.27	
74	-0.46	-0.52	-0.51	
75	-0.49	-0.61	-0.65	
76	-0.33	-1.32	-0.58	
77	-0.08	-0.02	-0.37	
78	-0.21	-0.51	0.50	
79	0.82	-3.99	-4.13	
80	-0.20	-0.09	-0.43	
81	-0.32	0.01	-0.13	
82	-0.60	-0.17	-0.14	
83	-0.58	-0.37	-0.16	
84	-0.09	0.61	0.60	
85	-0.06	0.72	-0.31	
86	0.16	2.43	0.30	
87	-0.02	0.20	-1.36	
88	0.26	1.06	-1.19	
89	0.44	-0.24	-1.21	
90	0.18	-0.04	-0.28	
91	0.19	-0.16	-0.54	
92	0.65	0.09	-0.27	
93	0.73	0.03	-0.55	
94	0.63	-0.51	-1.64	

n.d., not determined

GCDH R88C

	ΔTm (°C)			
Compound	10 µM	30 µM	100 µM	
1	-0.06	-0.10	-0.02	
2	0.07	0.17	0.15	
3	1.20	2.84	2.84	
4	0.52	0.50	0.83	
5	0.26	0.10	0.30	
6	0.22	0.72	1.73	
7	0.83	0.27	1.05	
8	-0.25	-0.34	0.03	
9	-0.14	-0.16	-0.16	
10	0.08	1.05	n.d.	
11	0.51	0.80	0.91	
12	0.34	0.40	-0.67	
13	0.06	0.42	0.28	
14	0.21	0.35	0.65	
15	0.64	1.19	0.44	
16	0.04	0.46	0.48	
17	-0.05	-0.26	-0.08	
18	-0.04	-0.12	-0.14	
19	4.19	2.52	1.11	
20	-0.36	-0.44	n.d.	
21	0.44	0.35	0.72	
22	0.23	0.05	-0.17	
23	0.10	-0.01	0.13	
24	0.00	0.03	0.34	
25	1.72	2.55	2.18	
26	0.43	0.62	0.73	
27	0.26	0.43	0.61	
28	0.00	0.34	0.41	
29	0.76	0.16	1.41	
30	-0.36	-0.41	0.87	
31	0.10	-0.06	0.51	
32	0.16	-0.10	0.56	
33	0.53	-0.35	1.86	
34	2.46	3.62	n.d.	
35	3.71	4.36	4.37	
36	0.53	0.83	n.d.	
37	0.63	-0.25	0.51	
38	0.53	0.83	0.75	
39	0.18	0.19	0.39	
40	0.17	0.12	-0.27	
41	0.46	1.14	2.68	
42	0.68	0.39	n.d.	
43	0.77	0.30	0.41	
44	0.55	0.29	0.37	
45	0.43	0.97	-0.49	
46	1.58	1.66	-0.64	
47	2.58	2.31	1.30	
48	1.27	0.50	1.43	
49	1.21	2.92	2.31	

	ΔTm (°C)			
Compound	10 µM	30 µM	100 µM	
50	0.46	1.80	n.d.	
51	1.25	1.81	2.90	
52	-0.63	2.16	3.49	
53	0.20	1.27	2.10	
54	1.45	1.01	0.36	
55	2.12	2.14	2.16	
56	0.60	0.20	1.18	
57	3.02	2.54	1.68	
58	-0.07	2.42	3.39	
59	1.56	1.33	0.76	
60	n.d.	-4.61	-11.96	
61	2.29	2.79	2.53	
62	1.14	1.60	1.70	
63	2.69	2.71	2.36	
64	0.38	0.39	0.96	
65	0.77	1.09	2.22	
66	2.35	1.08	1.79	
67	2.82	10.00	1.10	
68	1.35	1.64	2.11	
69	1.74	2.29	2.63	
70	1.04	1.11	1.40	
71	2.15	2.36	4.26	
72	0.31	0.45	0.13	
73	1.35	1.32	0.84	
74	0.06	0.19	0.09	
75	0.09	0.11	0.03	
76	1.49	0.81	n.d.	
77	0.98	1.42	2.22	
78	0.54	1.15	0.89	
79	3.46	1.97	n.d.	
80	1.06	0.66	1.42	
81	0.79	1.54	1.04	
82	-0.37	-0.27	n.d.	
83	-0.06	-0.09	0.17	
84	1.39	1.15	1.28	
85	2.35	2.25	0.44	
86	1.19	1.38	1.25	
87	1.51	0.46	0.27	
88	0.63	0.15	n.d.	
89	-2.22	-2.08	-1.14	
90	-1.13	-0.08	-0.54	
91	-1.86	0.06	0.66	
92	-1.56	-1.48	-1.48	
93	-1.21	-1.32	-0.81	
94	-2.16	-0.77	-1.69	

n.d., not determined

GCDH V400M

Compound	ΔTm (°C)		
	10 μM	30 μM	100 μM
1	0.12	0.11	0.35
2	-0.42	0.01	0.01
3	-0.01	-2.33	n.d.
4	1.04	1.34	2.03
5	0.57	1.34	1.81
6	0.62	1.02	1.26
7	-0.37	-0.29	-0.39
8	-0.31	-0.35	-0.20
9	0.25	0.58	0.37
10	-0.03	-0.06	n.d.
11	0.07	0.13	-0.34
12	1.27	1.30	-0.23
13	0.81	0.71	0.55
14	0.55	0.67	1.17
15	-0.25	-0.11	-0.30
16	-0.35	0.19	0.43
17	0.38	0.60	0.71
18	0.01	0.16	0.14
19	1.59	3.42	1.43
20	1.32	1.45	n.d.
21	1.07	1.29	1.69
22	0.80	0.41	0.57
23	-0.47	-0.09	-0.38
24	-0.68	-0.41	-0.51
25	-0.86	-0.26	-0.29
26	0.27	0.21	-0.03
27	-0.13	0.06	-0.02
28	-0.34	-0.20	-0.29
29	-0.89	-0.25	0.15
30	-0.86	0.69	0.58
31	-0.59	-0.22	-0.19
32	-0.54	-0.31	-0.41
33	-0.68	-0.45	-0.30
34	2.32	2.99	3.47
35	3.90	3.47	2.87
36	-0.13	0.00	-0.22
37	-0.66	-0.71	-0.59
38	-0.98	0.15	0.32
39	-0.12	-0.64	-0.92
40	-0.39	-0.72	-1.32
41	0.51	0.01	0.51
42	0.16	-0.14	-0.60
43	0.10	-0.21	-0.36
44	-0.23	-0.50	-1.51
45	-0.48	-0.40	0.16
46	-0.87	-0.47	0.15
47	-0.72	0.00	1.02
48	0.08	0.24	0.76
49	0.35	0.95	2.60
50	-0.24	-0.29	-0.76
51	-0.22	0.01	1.05
52	-0.48	-0.48	0.81
53	-0.36	-0.40	-0.38
54	-0.65	-0.99	-1.65
55	-0.23	-0.36	-0.40
56	0.63	0.26	0.47
57	0.03	-0.02	n.d.
58	0.43	1.05	3.04
59	-0.66	-0.66	-0.75
60	n.d.	-0.62	n.d.
61	-0.05	0.48	1.57
62	-0.40	-0.29	-0.53
63	-0.38	-0.56	-0.27
64	0.76	0.59	0.90
65	0.68	1.57	3.17
66	0.40	0.68	1.91
67	0.63	1.64	1.63
68	-0.01	0.74	2.38
69	-0.76	-0.32	-0.13
70	1.51	1.85	1.95
71	1.14	2.76	4.18
72	0.04	0.18	0.16
73	-1.16	-0.82	-1.30
74	-0.73	-0.11	0.11
75	-0.19	-0.21	-0.08
76	-0.86	-1.12	n.d.
77	0.63	1.20	2.17
78	0.85	0.87	0.85
79	1.33	1.33	n.d.
80	0.08	-0.06	0.22
81	-0.63	-0.48	-0.25
82	0.04	0.14	0.86
83	0.12	0.44	0.70
84	-0.15	0.37	1.06
85	-0.17	-0.13	0.07
86	1.06	1.07	1.10
87	0.72	0.76	1.00
88	0.39	0.39	2.01
89	-0.16	0.21	0.11
90	-0.65	-0.13	-0.17
91	-0.77	-0.16	-0.09
92	0.69	1.03	0.86
93	0.3	0.73	0.82
94	-0.04	0.17	n.d.

n.d., not determined

GCDH E414K – ΔTm for the first TSA transition midpoint (ΔTm1)

	ΔTm1 (°C)			
Compound	10 µM	30 µM	100 µM	
1	-0.06	-0.04	-0.17	
2	-35.23	-35.24	-35.24	
3	-0.51	n.d.	n.d.	
4	0.16	0.31	0.15	
5	0.22	0.29	-0.08	
6	0.34	0.82	1.13	
7	0.15	0.18	-0.05	
8	0.02	0.19	0.09	
9	-0.09	-0.33	-0.31	
10	-0.10	n.d.	n.d.	
11	0.26	-0.31	-3.08	
12	1.96	14.55	6.41	
13	0.35	0.32	0.37	
14	0.10	0.39	0.43	
15	0.51	-0.17	0.15	
16	-0.48	-0.40	0.50	
17	-0.36	-0.32	-0.50	
18	0.24	-0.17	0.26	
19	1.35	9.04	-1.07	
20	-0.85	0.83	n.d.	
21	0.02	0.44	0.40	
22	0.23	0.12	0.02	
23	-0.04	0.00	n.d.	
24	0.14	0.04	0.22	
25	0.24	0.24	0.73	
26	0.46	n.d.	n.d.	
27	0.24	-0.02	0.37	
28	0.34	0.97	0.89	
29	0.36	n.d.	-1.76	
30	0.22	n.d.	n.d.	
31	n.d.	n.d.	1.27	
32	0.30	0.50	0.43	
33	0.37	0.54	n.d.	
34	0.62	0.75	1.06	
35	2.75	3.11	1.70	
36	0.79	1.30	-0.14	
37	0.33	0.11	0.20	
38	0.31	-0.34	-0.78	
39	0.15	-0.07	-0.12	
40	0.29	0.10	-0.39	
41	-0.41	0.35	-0.49	
42	0.22	0.25	n.d.	
43	0.33	0.28	0.27	
44	0.40	0.38	-0.02	
45	-0.05	-0.13	0.02	
46	0.05	0.11	0.22	
47	0.15	0.33	0.46	
48	0.14	0.29	0.35	
49	0.00	0.33	n.d.	

	ΔTm1 (°C)			
Compound	10 µM	30 µM	100 µM	
50	0.19	0.39	n.d.	
51	0.05	0.28	0.56	
52	-0.03	0.23	-1.37	
53	0.00	-0.02	0.25	
54	0.16	0.10	-0.21	
55	0.05	0.07	0.04	
56	0.33	0.47	0.43	
57	-0.02	n.d.	n.d.	
58	0.79	3.22	n.d.	
59	0.31	0.26	0.27	
60	n.d.	n.d.	n.d.	
61	-0.46	0.13	1.28	
62	0.31	0.21	0.04	
63	0.43	0.40	0.23	
64	0.35	0.60	1.35	
65	0.50	0.62	0.89	
66	1.48	2.26	4.78	
67	-0.21	-0.57	-1.17	
68	0.34	0.34	0.51	
69	0.36	0.31	0.36	
70	0.95	0.47	n.d.	
71	0.79	0.79	1.76	
72	0.32	0.39	0.50	
73	0.19	0.22	0.11	
74	0.10	-0.03	0.08	
75	-0.04	0.05	-0.19	
76	0.37	0.59	n.d.	
77	0.56	0.68	0.14	
78	0.17	0.27	0.30	
79	n.d.	n.d.	n.d.	
80	0.20	0.52	1.11	
81	0.07	0.33	0.33	
82	0.20	0.13	n.d.	
83	0.05	-0.11	-0.25	
84	0.40	0.48	0.64	
85	0.42	0.38	0.38	
86	0.25	0.43	0.14	
87	0.52	0.55	0.78	
88	0.63	0.65	n.d.	
89	0.19	0.26	0.37	
90	0.18	0.26	0.38	
91	0.40	0.33	0.73	
92	0.35	0.21	0.36	
93	0.23	0.36	0.52	
94	0.18	1.10	n.d.	

n.d., not determined

GCDH E414K – ΔTm for the second TSA transition midpoint (ΔTm2)

	ΔTm2 (°C)		
Compound	10 µM	30 µM	100 µM
1	-0.18	-0.21	-0.13
2	0.00	0.02	-0.05
3	0.18	n.d.	n.d.
4	0.44	0.76	1.36
5	0.33	0.32	0.47
6	-0.14	0.27	0.09
7	-0.24	-0.02	0.14
8	-0.25	-0.10	-0.11
9	-0.29	1.05	-0.03
10	-0.23	0.14	n.d.
11	0.82	0.42	0.14
12	-0.13	-0.44	-0.93
13	0.14	-0.14	0.32
14	-0.02	0.13	0.58
15	0.48	2.14	0.43
16	-0.30	0.03	0.07
17	0.12	0.13	0.14
18	-0.12	0.47	-0.60
19	0.90	1.95	1.66
20	2.17	0.89	n.d.
21	0.62	0.08	-0.02
22	0.22	0.65	0.13
23	-0.31	-0.29	0.58
24	-0.16	0.09	0.21
25	0.27	1.22	0.80
26	0.28	0.44	-0.14
27	0.02	0.21	0.25
28	-0.30	-0.28	-0.05
29	0.16	1.33	1.52
30	-0.26	-0.36	0.08
31	0.14	-1.25	-0.23
32	-0.14	-0.31	-0.08
33	0.16	-0.08	0.34
34	0.36	0.92	0.75
35	0.21	0.90	0.01
36	0.01	0.04	-0.12
37	-0.10	0.29	0.54
38	-0.07	0.03	-0.30
39	-0.39	-0.21	-0.17
40	-0.42	-0.42	-0.45
41	0.40	1.04	2.92
42	0.37	0.26	1.19
43	0.12	0.05	0.19
44	0.12	0.02	0.01
45	-0.29	-0.29	-0.09
46	-0.10	0.05	0.06
47	0.09	0.33	0.52
48	0.60	0.60	0.86
49	1.47	1.89	3.06

	ΔTm2 (°C)		
Compound	10 µM	30 µM	100 µM
50	-0.10	-0.21	-0.23
51	1.64	1.61	1.57
52	-0.20	-0.04	0.29
53	-0.22	-0.16	-1.10
54	0.03	-0.30	-0.52
55	0.58	0.76	0.60
56	0.60	0.77	0.69
57	0.46	0.50	n.d.
58	0.58	0.71	n.d.
59	0.01	0.12	-0.26
60	-4.38	n.d.	n.d.
61	0.13	0.47	-0.48
62	0.01	-0.06	-0.29
63	0.15	0.13	-0.01
64	0.70	0.65	0.68
65	0.63	0.81	1.21
66	-0.25	-0.52	-0.47
67	0.61	0.91	0.32
68	-0.42	0.38	0.70
69	-0.16	0.51	0.93
70	0.01	0.45	0.90
71	0.37	0.48	0.24
72	-0.22	0.26	0.17
73	-0.41	0.19	-0.02
74	-0.36	0.20	0.26
75	-0.20	0.03	-0.20
76	-0.12	-0.01	n.d.
77	0.47	0.44	1.12
78	0.58	0.48	0.05
79	n.d.	n.d.	n.d.
80	1.31	0.53	0.17
81	1.42	0.39	0.31
82	-0.08	0.02	0.48
83	-0.37	-0.01	-0.08
84	0.13	-0.04	0.01
85	0.11	0.23	0.11
86	0.33	0.31	0.48
87	0.03	0.30	-0.21
88	0.26	-0.20	n.d.
89	-0.27	-0.19	-0.36
90	0.12	0.22	0.66
91	-0.01	0.32	0.35
92	0.49	0.61	0.69
93	1.05	1.21	1.53
94	-0.30	0.10	n.d.

n.d., not determined

GCDH A433E

	ΔTm (°C)			
Compound	10 µM	30 µM	100 µM	
1	-0.23	-0.44	-0.34	
2	1.05	-0.08	0.55	
3	1.00	1.27	2.31	
4	0.26	0.67	1.04	
5	-0.08	0.01	0.90	
6	-0.46	-0.15	0.20	
7	0.16	-0.65	0.89	
8	0.25	-0.47	-0.17	
9	-0.44	-0.23	-0.32	
10	0.87	0.49	n.d.	
11	1.22	0.04	1.73	
12	0.03	0.19	0.55	
13	0.04	0.29	0.05	
14	-0.13	0.40	0.80	
15	0.41	-0.61	0.35	
16	-0.66	-0.45	0.29	
17	-0.42	-0.34	-0.17	
18	0.26	0.10	0.13	
19	3.37	6.13	8.82	
20	0.09	-0.29	n.d.	
21	0.06	0.13	0.68	
22	-0.04	-0.25	-0.29	
23	0.60	0.06	0.56	
24	1.12	1.25	1.28	
25	2.07	3.17	2.71	
26	0.82	0.39	0.83	
27	0.91	0.83	0.92	
28	-0.25	0.41	1.19	
29	-0.32	1.33	1.7	
30	-0.17	0.47	2.15	
31	0.2	0.36	0.36	
32	0.7	1.12	1.62	
33	1.32	1.5	2.24	
34	0.94	2.1	2.68	
35	1.96	3.41	3.68	
36	0.52	0.92	1.5	
37	0.72	1.2	0.98	
38	0.49	1.14	1.17	
39	-0.05	-0.06	0.17	
40	0.95	0.54	0.44	
41	1.79	2.15	3.41	
42	0.44	0.25	1.22	
43	0.39	0.11	0.1	
44	0.75	-0.03	0.06	
45	-0.26	-0.19	-0.17	
46	-0.23	0.66	1.56	
47	-0.02	0.8	1.6	
48	0.09	0.08	0.36	
49	0.25	0.84	1.92	

	ΔTm (°C)			
Compound	10 µM	30 µM	100 µM	
50	-0.33	0.33	3.48	
51	-0.42	1.23	2.24	
52	-0.62	0.57	2.86	
53	-0.23	-0.24	0.53	
54	-0.25	-0.93	0.51	
55	3.87	3.10	3.51	
56	-0.16	0.09	0.55	
57	1.54	0.69	n.d.	
58	0.12	0.37	0.01	
59	-0.87	1.50	1.64	
60	n.d.	0.10	n.d.	
61	1.03	1.05	1.35	
62	0.23	0.01	-0.04	
63	0.67	0.49	0.38	
64	0.16	0.26	0.59	
65	0.40	0.57	1.10	
66	0.22	1.09	2.01	
67	1.08	1.29	1.42	
68	0.56	0.24	0.69	
69	1.36	0.89	1.11	
70	0.82	0.90	1.21	
71	0.49	1.25	2.48	
72	-0.15	-0.05	0.07	
73	0.80	0.02	0.69	
74	-0.23	-0.32	0.17	
75	-0.26	-0.26	-0.22	
76	0.90	1.06	n.d.	
77	0.87	0.82	1.06	
78	0.46	0.44	0.45	
79	2.08	1.93	n.d.	
80	0.26	0.21	0.54	
81	1.23	0.59	1.06	
82	-0.14	-0.43	0.38	
83	-0.36	-0.31	-0.17	
84	0.08	0.03	0.05	
85	0.36	0.29	0.19	
86	n.d.	-0.60	-0.17	
87	1.00	1.06	1.03	
88	0.41	0.27	0.95	
89	0.34	0.47	0.47	
90	0.81	0.77	0.72	
91	1.76	0.85	0.88	
92	0.63	0.83	0.31	
93	0.43	0.61	0.68	
94	0.88	0.80	1.01	

n.d., not determined

Table S4. Library B Screen Thermal Shift Assay (76 compounds). The thermal shift observed between the assay melting points of GCDH wild-type and three GCDH variants (R88C, V400M, and A433E) in the presence and absence of compound (ΔTm) is shown for each compound at 3 different concentrations. Graphical representations on the rightmost column depict the transition of the ΔTm for the 3 concentrations in ascending order (minimum [0°C] and maximum [3°C] ΔTm values were set).

GCDH WT

	ΔTm (°C)			
Compound	10 µM	30 µM	100 µM	
1	0.14	0.14	-0.26	
2	2.55	3.24	n.d.	
3	0.20	0.04	1.02	
4	0.31	0.41	0.39	
5	0.57	0.63	0.80	
6	0.21	0.33	0.29	
7	0.09	0.15	0.04	
8	-0.31	0.04	-0.18	
9	-0.04	0.04	0.07	
10	0.08	-0.01	0.11	
11	0.14	0.21	0.75	
12	0.58	1.00	0.42	
13	0.15	0.25	0.27	
14	0.49	0.94	2.00	
15	0.38	1.26	2.00	
16	-0.29	-0.10	0.19	
17	0.08	0.05	0.03	
18	0.09	0.07	0.28	
19	0.30	0.15	0.00	
20	0.68	0.28	0.47	
21	0.43	0.22	0.41	
22	0.26	0.26	0.20	
23	-0.32	-0.18	-0.14	
24	-0.26	-0.23	0.18	
25	0.09	0.08	0.03	
26	0.33	-0.06	0.30	
27	0.22	0.28	0.40	
28	0.10	0.32	0.72	
29	n.d.	n.d.	1.85	
30	2.39	0.50	n.d.	
31	1.76	2.07	-0.29	
32	0.01	0.00	0.23	
33	0.53	0.31	0.04	
34	0.18	0.52	-0.12	
35	0.17	0.17	0.10	
36	0.21	0.25	0.26	
37	n.d.	n.d.	2.05	
38	-1.73	-1.81	-1.80	
39	-0.06	-0.10	-0.06	
40	n.d.	n.d.	n.d.	

	ΔTm (°C)			
Compound	10 µM	30 µM	100 µM	
41	0.46	0.52	0.82	
42	0.28	0.33	0.06	
43	0.51	0.26	0.05	
44	0.30	0.23	0.09	
45	-0.21	-0.07	0.23	
46	-0.49	-0.49	-0.37	
47	-0.40	-0.07	-0.83	
48	0.60	0.42	0.67	
49	0.90	2.53	2.95	
50	0.36	0.66	0.48	
51	-0.71	-0.18	-0.71	
52	-0.47	-0.34	-0.35	
53	0.15	0.39	1.62	
54	-0.07	-0.17	0.30	
55	-0.44	-0.37	-0.50	
56	4.17	4.12	3.72	
57	0.12	0.42	0.38	
58	0.19	0.28	0.16	
59	-0.28	-0.59	-0.96	
60	0.06	0.06	0.27	
61	0.43	0.16	0.07	
62	0.63	-0.34	0.08	
63	-0.27	-0.35	-0.23	
64	1.68	1.50	1.45	
65	0.43	0.40	0.38	
66	0.36	0.24	0.29	
67	-0.38	0.25	0.44	
68	-0.82	-0.08	-0.06	
69	-0.56	-0.09	0.00	
70	0.84	1.37	1.83	
71	0.09	0.46	0.85	
72	-0.01	0.69	0.85	
73	-1.25	-3.65	-0.07	
74	-0.58	-1.64	0.23	
75	0.01	0.03	0.16	
76	0.21	0.08	0.10	

n.d., not determined

GCDH R88C

	ΔTm (°C)			
Compound	10 µM	30 µM	100 µM	
1	-0.26	-0.20	-0.40	
2	-1.65	-1.59	-1.18	
3	-1.72	-0.59	-0.51	
4	3.44	2.39	3.60	
5	0.72	2.10	2.14	
6	1.28	1.91	2.29	
7	-1.23	-1.33	-0.50	
8	1.04	-0.59	0.15	
9	0.48	0.52	-0.03	
10	-1.11	-0.73	-1.40	
11	-1.16	-1.65	-1.75	
12	n.d.	3.96	4.34	
13	2.55	2.30	0.86	
14	1.75	2.64	-0.61	
15	-0.86	-0.28	1.50	
16	1.55	2.82	0.46	
17	-0.99	-2.37	-2.44	
18	-0.58	-0.06	1.84	
19	-1.26	-0.06	0.11	
20	-0.24	-1.15	-1.02	
21	1.34	1.21	0.96	
22	1.49	1.83	1.54	
23	0.51	1.34	1.30	
24	-0.86	-0.84	-2.27	
25	-1.06	-0.38	-0.60	
26	1.37	1.62	1.63	
27	0.55	0.92	1.22	
28	1.15	1.28	1.47	
29	1.16	1.30	n.d.	
30	1.90	0.61	0.07	
31	1.83	1.37	n.d.	
32	0.00	-0.04	-0.12	
33	-0.37	-0.80	0.02	
34	4.48	4.00	-1.37	
35	1.29	0.69	0.02	
36	1.42	1.19	-0.68	
37	-1.23	-2.01	-2.73	
38	2.18	1.84	-1.40	
39	-1.06	-0.92	-0.87	
40	n.d.	n.d.	n.d.	

	ΔTm (°C)			
Compound	10 µM	30 µM	100 µM	
41	0.73	-0.49	0.48	
42	-0.54	-1.03	-0.41	
43	-0.75	-1.07	-1.26	
44	-1.02	-0.95	-1.33	
45	-0.05	0.15	1.08	
46	0.46	-0.20	0.66	
47	1.85	2.79	3.49	
48	0.22	0.13	-0.42	
49	2.21	2.04	2.05	
50	1.76	2.21	2.63	
51	1.70	1.92	1.77	
52	0.60	0.62	1.87	
53	1.51	1.28	1.84	
54	2.00	1.55	2.72	
55	1.94	2.16	2.98	
56	2.92	3.00	2.37	
57	0.10	0.28	1.27	
58	0.03	0.08	0.18	
59	0.17	-0.07	1.20	
60	0.04	-0.30	0.26	
61	-0.22	-0.10	-0.32	
62	-0.07	0.09	-0.02	
63	0.17	0.12	-0.04	
64	1.15	0.43	0.37	
65	-0.19	-0.02	0.03	
66	0.01	0.27	-0.10	
67	-0.09	-0.14	-0.23	
68	0.00	-0.01	-0.19	
69	1.05	-0.10	-0.21	
70	-0.07	0.08	0.06	
71	-0.01	0.23	0.45	
72	-0.42	-0.25	-0.22	
73	0.02	-0.28	-0.72	
74	-0.57	-0.48	-0.59	
75	-0.28	-0.34	-0.07	
76	0.12	-0.47	0.09	

n.d., not determined

GCDH V400M

	ΔTm (°C)			
Compound	10 μM	30 μM	100 μM	
1	-0.71	-0.19	-1.02	
2	2.49	-3.32	n.d.	
3	-1.67	-2.34	-1.53	
4	0.06	-0.14	-2.67	
5	0.98	-0.41	-0.08	
6	-1.70	-0.81	-0.53	
7	-0.07	-0.04	-0.99	
8	0.94	-0.08	-1.86	
9	-0.05	-0.43	-1.33	
10	-0.11	-1.09	-0.94	
11	-0.08	-0.52	-2.31	
12	4.61	3.68	3.81	
13	-1.01	0.44	-0.33	
14	-0.56	1.17	3.81	
15	0.77	2.88	3.41	
16	-0.54	1.37	-0.01	
17	-0.24	0.14	-0.10	
18	0.13	-0.14	-0.26	
19	1.42	1.97	-0.26	
20	5.40	5.80	7.00	
21	-0.45	-0.72	-1.08	
22	0.01	0.84	-0.15	
23	-0.78	0.65	-0.65	
24	-1.78	-0.13	-1.77	
25	-1.98	0.20	-0.86	
26	1.26	0.05	-10.57	
27	0.88	-0.99	-1.17	
28	0.60	0.07	0.11	
29	0.21	1.91	n.d.	
30	-3.57	n.d.	n.d.	
31	-1.29	-2.92	n.d.	
32	1.50	-1.75	-2.26	
33	-0.11	-0.52	-0.44	
34	3.97	2.43	2.64	
35	-1.17	0.00	-2.72	
36	0.45	-0.40	-0.22	
37	n.d.	n.d.	n.d.	
38	-1.71	-0.98	-1.96	
39	-1.26	-1.02	-1.21	
40	n.d.	n.d.	n.d.	

	ΔTm (°C)			
Compound	10 μM	30 μM	100 μM	
41	-0.19	-0.10	-0.02	
42	2.21	3.10	4.65	
43	-0.88	-0.94	-0.03	
44	-0.52	0.23	0.08	
45	-0.30	0.51	0.99	
46	-1.30	-0.15	0.36	
47	1.81	2.67	2.03	
48	-0.50	0.00	-0.35	
49	1.01	1.81	1.64	
50	0.35	0.64	1.94	
51	1.44	1.68	1.88	
52	-0.08	0.39	0.61	
53	0.49	-0.19	2.00	
54	-0.26	1.19	2.78	
55	1.96	2.05	3.59	
56	4.65	4.79	3.93	
57	0.10	0.50	1.30	
58	-0.32	-0.35	0.12	
59	0.39	0.83	0.97	
60	-0.32	-0.75	-0.18	
61	-0.57	-0.58	-0.59	
62	0.85	0.26	-0.48	
63	1.14	1.01	1.14	
64	-0.05	0.13	-0.48	
65	-0.12	-0.11	-0.16	
66	-0.31	-0.17	-0.24	
67	0.74	0.86	0.39	
68	0.59	0.55	0.10	
69	0.65	0.29	0.84	
70	0.92	0.71	0.40	
71	0.79	0.62	0.47	
72	0.45	0.35	0.53	
73	0.64	0.47	0.36	
74	0.16	0.19	0.01	
75	0.27	0.03	0.40	
76	0.73	1.83	1.22	

n.d., not determined

GCDH A433E

Compound	ΔTm (°C) 10 µM	30 µM	100 µM
1	-0.42	0.16	-0.08
2	1.26	2.24	n.d.
3	0.42	-0.12	0.88
4	0.66	0.56	0.36
5	0.45	0.82	1.41
6	0.07	0.26	0.32
7	1.03	-0.47	0.07
8	-0.09	-0.53	-0.21
9	0.02	0.07	-0.33
10	0.22	0.53	-0.41
11	1.15	2.09	2.03
12	0.75	0.30	0.27
13	-0.03	0.44	0.24
14	-0.36	0.27	1.66
15	0.43	1.45	2.47
16	0.00	0.31	0.22
17	-0.24	0.08	-0.03
18	0.34	0.35	0.29
19	1.74	0.48	-0.20
20	0.46	0.52	0.31
21	0.33	0.17	0.22
22	0.18	0.33	0.01
23	-0.16	-0.16	-0.29
24	0.17	0.02	-0.10
25	0.18	-0.46	0.30
26	0.45	0.38	-1.43
27	0.54	1.05	0.32
28	0.13	0.43	0.42
29	3.92	3.29	2.43
30	3.46	2.16	n.d.
31	2.36	2.40	1.14
32	0.24	0.32	0.15
33	0.08	0.22	0.19
34	1.22	0.67	0.41
35	0.15	0.33	-0.09
36	0.38	0.61	0.41
37	-0.72	0.43	0.69
38	-0.50	-0.22	-0.67
39	0.03	0.06	0.08
40	n.d.	n.d.	n.d.

Compound	ΔTm (°C) 10 µM	30 µM	100 µM
41	0.68	0.89	1.13
42	1.26	0.97	0.44
43	0.27	0.17	-0.05
44	0.23	0.52	0.01
45	0.03	0.09	0.90
46	-0.24	-0.07	2.06
47	2.63	3.00	2.05
48	0.61	0.46	0.18
49	1.93	2.35	2.12
50	1.31	1.87	1.50
51	2.24	1.76	0.98
52	0.21	0.57	1.00
53	0.66	1.14	1.64
54	1.01	1.99	2.26
55	2.43	2.79	2.61
56	5.15	4.36	4.27
57	0.33	0.86	1.04
58	0.17	0.33	0.36
59	0.04	1.03	1.42
60	0.01	0.14	0.24
61	-0.40	-0.18	-0.20
62	0.09	-0.11	0.02
63	0.77	0.71	0.30
64	0.38	0.39	-0.44
65	0.36	0.28	0.12
66	0.16	0.37	0.14
67	0.18	0.34	0.00
68	0.12	0.29	0.26
69	0.27	-0.05	-0.08
70	0.78	-0.02	1.15
71	0.65	0.37	0.71
72	0.11	0.29	0.26
73	0.03	0.08	0.11
74	0.02	0.26	0.14
75	-0.03	-0.02	0.06
76	0.08	0.27	-0.09

n.d., not determined

Table S5. Chemical properties of the final 25-compound library. All properties were predicted by CDDVault. Reference: Chemical Properties Calculated by CDD Vault During Chemical Registration – CDD Support (collaborativedrug.com). Analysis of the selected hits revealed good physicochemical properties.

Compound	Mol. Wt. (g/mol)	cLog P	HBD	HBA	CDDV TPSA (Å²)	RTB	cLog S	cLog D	CDDV pKa	HAC
A19	282.130	3.9	2	4	54.0	2	-4.5	3.3	8.1	18
A25	320.130	2.2	3	4	61.4	3	-3.4	1.9	8.1	15
A29	249.097	2.1	3	4	61.4	3	-2.9	1.8	8.3	15
A34	318.423	2.7	1	4	58.5	2	-3.9	2.7	2.7	21
A35	339.829	2.0	1	5	71.4	2	-3.5	2.0	4.5	21
A41	310.716	1.4	2	6	76.0	4	-2.8	1.4	8.7	21
A49	340.433	3.8	1	5	78.7	5	-4.8	3.0	8.4	23
A52	419.437	2.4	2	8	106.4	8	-4.3	2.0	6.5	31
A55	351.793	4.0	2	5	71.1	4	-5.0	3.3	2.8	25
A57	326.179	3.6	2	4	58.6	5	-4.6	3.3	8.4	21
A58	465.509	5.1	3	7	115.2	9	-6.6	1.6	4.1	35
A66	492.443	5.6	2	9	124.4	4	-7.1	4.3	8.6	37
A71	387.202	2.9	2	5	74.8	4	-4.4	2.1	4.0	19
B12	262.312	3.3	1	3	42.0	3	-3.9	2.9	4.2	20
B14	363.389	2.4	1	5	61.8	2	-3.9	2.3	8.7	25
B15	301.371	1.5	1	5	61.8	3	-2.9	1.5	8.2	21
B29	332.190	5.3	2	4	54.0	2	-5.9	4.2	9.2	22
B30	332.190	5.1	2	4	54.0	2	-5.7	4.2	8.5	22
B31	316.575	4.2	2	4	54.0	2	-4.9	3.4	8.2	19
B34	260.724	3.4	1	3	42.0	3	-4.0	3.1	4.9	18
B37	305.377	3.7	1	3	38.3	5	-4.5	3.7	15.5	23
B40	417.368	5.3	1	5	67.8	5	-6.4	5.3	8.2	24
B47	339.782	3.7	3	5	77.2	4	-4.7	2.9	10.5	24
B49	314.366	4.2	3	5	82.4	3	-4.9	3.3	8.0	22
B56	281.142	4.8	2	3	41.1	2	-5.1	4.0	8.8	18

Chemical properties computed: (a) Lipinski and Veber properties: MW, Molecular weight (≤500 g/mol); cLog P, Calculated n-octanol/water partition coefficient (≤5); HBD, H-Bond Donors (sum of NH and OH ≤5); HBA, H-Bond Acceptors (sum of N and O ≤10); CDDV TPSA, Topological polar surface area (≤140); RTB, Rotatable Bonds (≤10), and (b) Additional properties: cLog S, Calculated aqueous solubility at pH 7.4; cLog D, Calculated distribution coefficient at pH 7.4; CDDV pKa, Calculated the strongest pKa (either basic or acidic); HAC, Heavy-atoms count (=all non-hydrogen atoms).

Table S6. ITC binding and thermodynamics parameters with binding compounds.

Compound	N (sites)	K_D (μM)	ΔH (kJ/mol)	ΔG (kJ/mol)	-TΔS (kJ/mol)
CrotCoA	0.4 ± 0.4	24.9 ± 17.5	-23.4 ± 25.7	-26.1	-2.7
A41	1.2 ± 0.2	8.2 ± 4.5	-3.7 ± 0.7	-28.6	-24.8
A49	0.8 ±0.6	3.4 ± 1.3	-21.4 ± 2.4	-30.7	-9.3
A58	1.2 ± 0.1	6.9 ± 2.5	-3.8 ± 0.4	-29.0	-25.2
A66	1*	44.9 ± 7.0	-19.2 ± 1.4	-24.4	-5.2
A71	0.7 ± 0.1	7.7 ± 1.3	-10.5 ± 0.9	-28.7	-18.2

Number of binding sites (N), predicted binding constant (KD), enthalpy (ΔH), Gibbs energy (ΔG) and entropy changes (-TΔS) are shown for the interaction between GCDH WT and each compound. * Number of binding sites was fixed as 1 prior to fitting. CrotCoA, crotonyl-CoA.

Table S7. SAR data of commercially available compounds with variations of scaffold 1. Summary of the structural variations that lead to protein stability and an activator profile of the compounds**.**

The analysis revealed phenylurea has the common structure among the validated hits. In the binding model, the two amines of urea establish hydrogen bonds. Group R1 comprises a hydrophobic core with, ideally, an atom or functional group that can form an extra hydrogen bond. Group X should contain one or two halogen atoms, preferably positioned in the meta and para positions, which, along with the benzene ring, establish hydrophobic interactions.

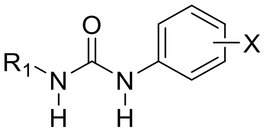

Compound	R1	X	Stability Effect (ΔTm score)	Relative Enzymatic Activity (% DMSO)	Screening General Evaluation
A19	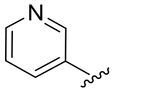	3,4-diCl	14	122 ± 2	Stabiliser. Activator
A25	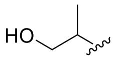	4-I	6	117 ± 7	Stabiliser. Activator
A29	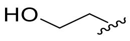	3,4-diCl	3	141 ± 11	Stabiliser. Activator
A41	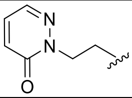	3-Cl, 4-F	10	135 ± 59	Stabiliser. Activator
B29	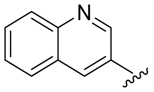	3,4-diCl	10	134 ± 4	Stabiliser. Activator
B30	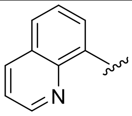	3,4-diCl	8	124 ± 8	Stabiliser. Activator
B31	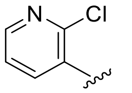	3,4-diCl	7	147 ± 5	Stabiliser. Activator
B56	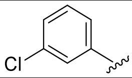	3-Cl	12	113 ± 5	Stabiliser. Activator

GCDH wild-type (150 nM) activity was determined in the presence of 10 µM of compound (in comparison to DMSO control); Stability effect, sum of ΔTm scores for the different GCDH variant proteins tested; ΔTm score, assigned based on the ΔTm (change in melting temperature) for each protein variant, with scores of 1 given for ΔTm > 1°C, 2 for ΔTm > 2°C and 4 for ΔTm > 3°C.

Table S8. SAR data of compounds available with variations of scaffold 1 compared to the corresponding parent compound selected as a screening hit. Structural variations (in red) that lead to a loss of stability are shown.

The table includes subtle variations of scaffold1 that challenge the established binding model. Among the compounds available, it was possible to study the effect of modifications such as: replacement of the oxygen in urea with sulphur (A61), removal of the amine hydrogens from urea (B2, B32), elongation of the linker of both arms (A23, B3), replacement of aromatic groups (A24, B33), alteration of positions without addition of halogens (A17, A18, A22, A26, A42, A80), and inclusion of branching (A62). Due to the loss of stability, these compounds were deprioritized for further testing (secondary assays). However, this structural information provides insight for future compound optimization.

Parent Compound	Compound	Chemical structure	Stability Effect (ΔTm score)
A19	A23	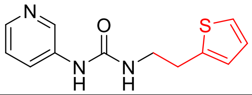	0
	A24	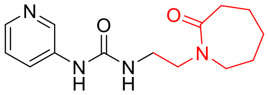	0
	B2	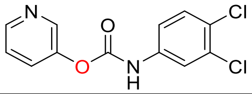	-2
	B3	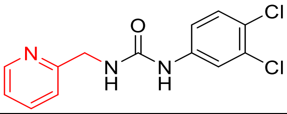	-8
A25	A17	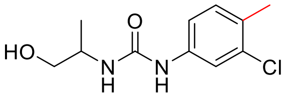	0
	A18	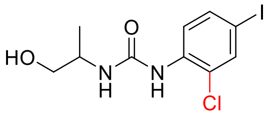	0
	A22	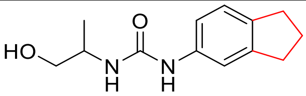	0
A29	A26	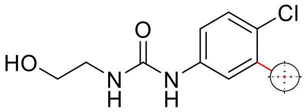	0
	A80	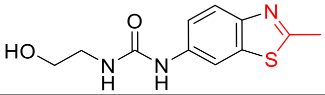	0
	A62	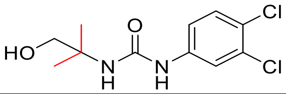	0
	A61	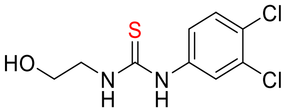	2
A41	A42	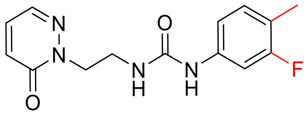	0

Parent Compound	Compound	Chemical structure	Stability Effect (ΔTm score)
NA	B32	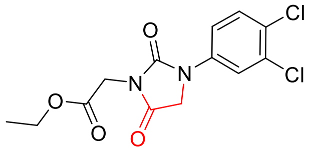	0
NA	B33	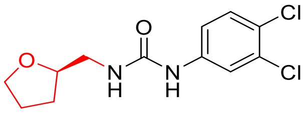	0

Stability effect, sum of ΔTm scores. Values were determined by TSA using the GCDH wild type (WT) and variants (R88C, V400M, and A433E). For assay details see Experimental Section. NA, Not available.

Table S9. Predicted binding mode of two representative compounds from scaffold 1 in the allosteric pocket. Docking studies in the monomeric unit of the crystal structure of GCDH (1SIQ), of compounds A19, a stabilizer, and A24, a non-stabilizer. Docking pose of scaffold 1 compounds showed similar conformations but distinct interaction patterns.

<table>
<tr><th>Docking Prediction Result</th><th>Docking Prediction Analysis</th></tr>
<tr><td>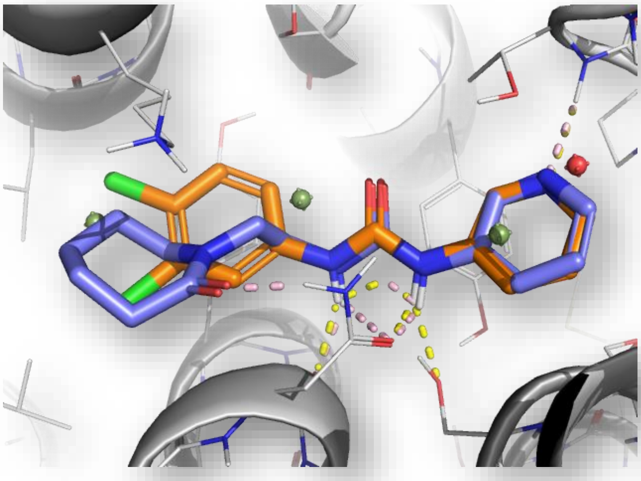
Docking prediction: compound A19 (orange) aligns with the pharmacophore (red and green spheres), indicating potential interactions and optimal distances within the identified allosteric pocket. Compound A24 (purple) does not overlap correctly. Hydrogen bond interactions are represented by yellow dashes (A19) and light pink lines (A24).</td><td>The prediction indicates that the nitrogen of the pyrimidine interacts with Trp109, and this aromatic ring also has partial pi-pi interactions with Tyr123 in both. The two NH groups of urea form hydrogen bonds with Asn292 and Ser146, although the latter is not present in compound A24. The most significant difference observed is the aromatic stacking interactions, where the difluoro phenyl ring establishes interactions with Tyr295 and its hydrophobic environment (Leu353 and Val367), which is not present in compound A24. In compound A24, the new R1 chain extends beyond the typical interaction plane, seeking alternative binding interactions.</td></tr>
<tr><td colspan="2">Summary: the binding mode studies highlight the relevance of appropriate groups, distances, orientations, and conformations to establish key interactions. A loss of these interactions can result in a loss of stabilisation effect or even destabilisation in the primary TSA.</td></tr>
</table>

Table S10. Solubility data of lead compounds. The solubility data of 5 lead compounds and control compound diclofenac sodium in 20 mM HEPES buffer containing 200 mM NaCl (pH 7.0) and PBS (pH 7.2), as calculated by LC-MS/MS analysis.

Compound ID	Solvent	Solubility (µM)
Diclofenac sodium	20 mM HEPES 200 mM NaCl (pH 7.0)	297.98
A49	20 mM HEPES 200 mM NaCl (pH 7.0)	0.51
A55	20 mM HEPES 200 mM NaCl (pH 7.0)	2.42
A71	20 mM HEPES 200 mM NaCl (pH 7.0)	70.37
B29	20 mM HEPES 200 mM NaCl (pH 7.0)	n.d.
B31	20 mM HEPES 200 mM NaCl (pH 7.0)	0.93
Diclofenac sodium	PBS pH 7.2	293.80
A49	PBS pH 7.2	0.52
A55	PBS pH 7.2	4.03
A71	PBS pH 7.2	248.94
B29	PBS pH 7.2	n.d.
B31	PBS pH 7.2	0.92

Note: Solubility values are shown as mean (n=2). Any value close to, or above, the upper limit (300 µM) indicates that the compound may have a solubility at, or above, the upper limit.
PBS, phosphate buffer saline. n.d., not detectable (no visible peak could be detected in the sample).

Figure S1. HPLC traces for lead compounds.

The purity of compounds was determined by LC-MS on Waters Alliance HT 2795 and PDA 2996 at a wavelength of 254 nM; mass spectrometer: Micromass ZQ2000 single quadrupole (ESI) using a SunFire C18 (100 mm x 2.1 mm, 3.5 µm) column under the following chromatography conditions: mobile phase A, 10 mM ammonium formate buffer pH 4 with HCOOH; Mobile phase B, water; Mobile phase C, acetonitrile; flow rate, 0.5 mL/min; elution gradient (A:B:C *v/v*), 5:85:10 (0–3 min), 5:85:10 to 5:10:85 (3–12 min), 5:10:85 (12–20 min).

Compound A49 - HPLC-MS

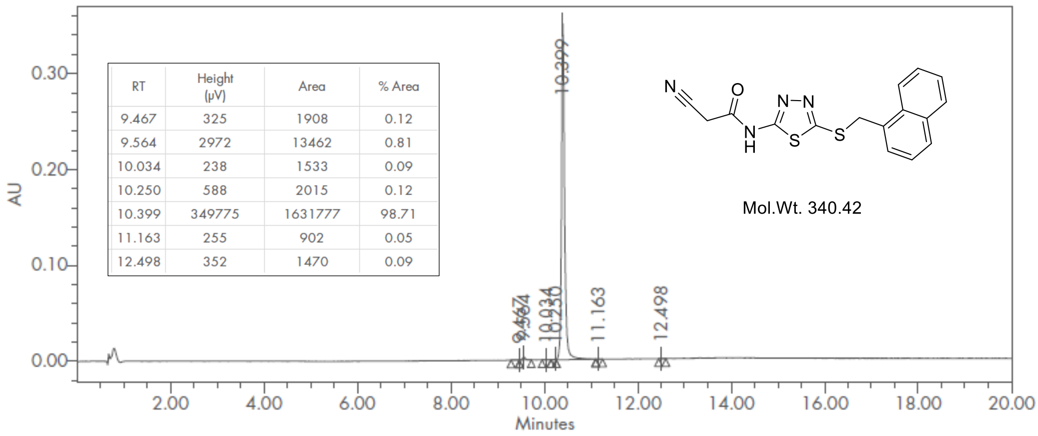

RT	Height (µV)	Area	% Area
9.467	325	1908	0.12
9.564	2972	13462	0.81
10.034	238	1533	0.09
10.250	588	2015	0.12
10.399	349775	1631777	98.71
11.163	255	902	0.05
12.498	352	1470	0.09

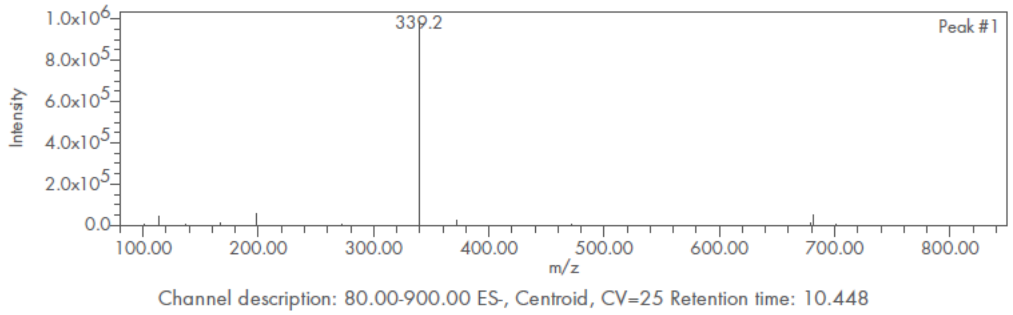

Compound A55 - HPLC-MS

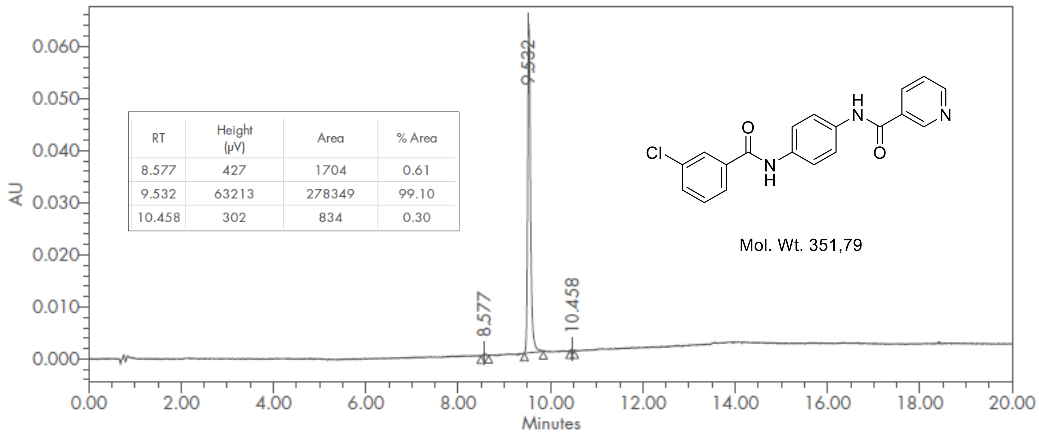

RT	Height (µV)	Area	% Area
8.577	427	1704	0.61
9.532	63213	278349	99.10
10.458	302	834	0.30

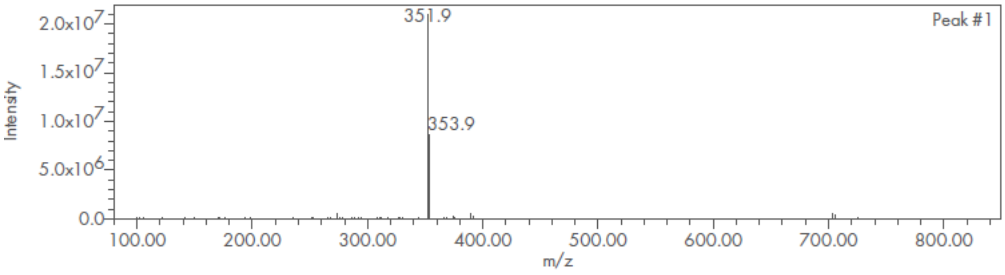

Channel description: 80.00-900.00 ES+, Centroid, CV=25 Retention time: 9.568

Compound A71 - HPLC-MS

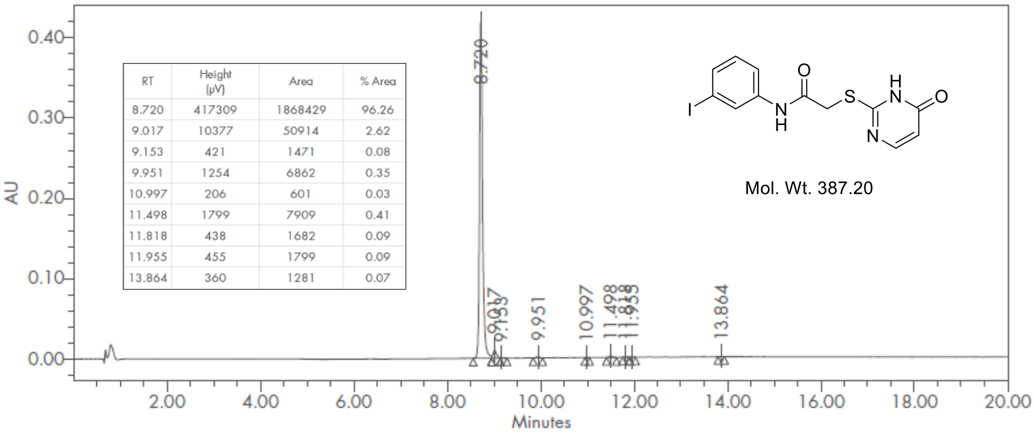

RT	Height (μV)	Area	% Area
8.720	417309	1868429	96.26
9.017	10377	50914	2.62
9.153	421	1471	0.08
9.951	1254	6862	0.35
10.997	206	601	0.03
11.498	1799	7909	0.41
11.818	438	1682	0.09
11.955	455	1799	0.09
13.864	360	1281	0.07

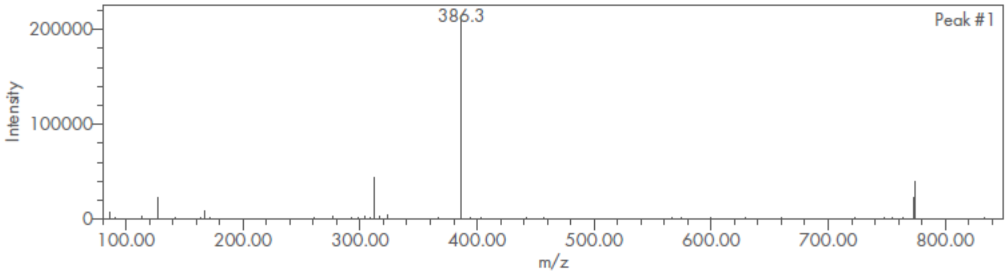

Channel description: 80.00-900.00 ES-, Centroid, CV=25 Retention time: 8.763

Compound B29 - HPLC-MS

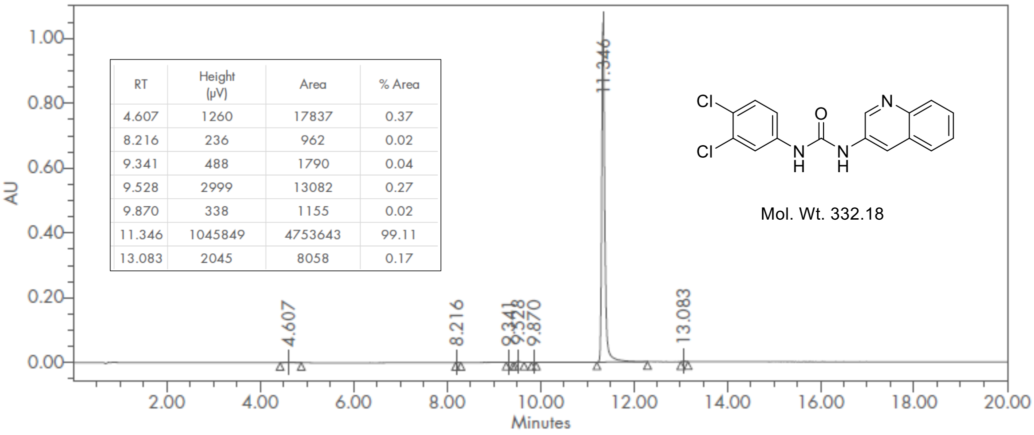

RT	Height (μV)	Area	% Area
4.607	1260	17837	0.37
8.216	236	962	0.02
9.341	488	1790	0.04
9.528	2999	13082	0.27
9.870	338	1155	0.02
11.346	1045849	4753643	99.11
13.083	2045	8058	0.17

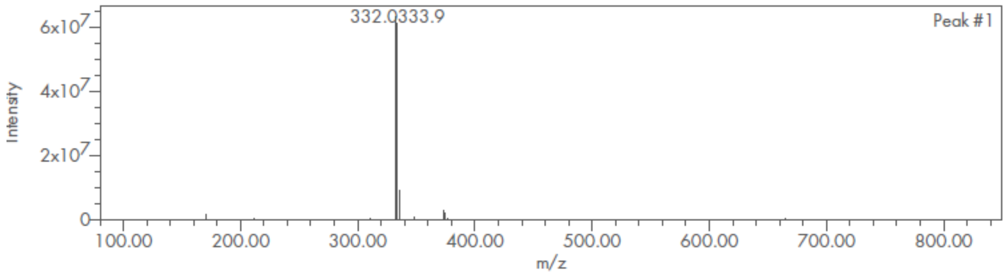

Channel description: 80.00-900.00 ES+, Centroid, CV=25 Retention time: 11.377

Compound B31 - HPLC-MS

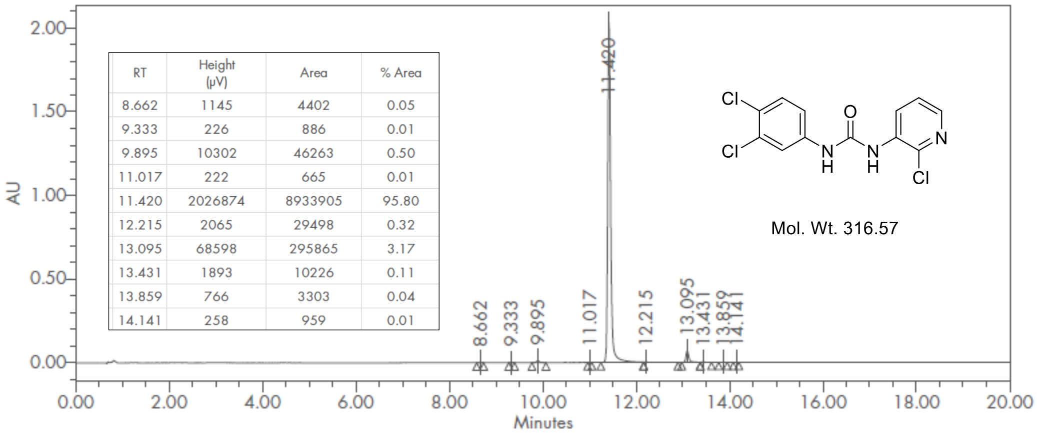

RT	Height (µV)	Area	% Area
8.662	1145	4402	0.05
9.333	226	886	0.01
9.895	10302	46263	0.50
11.017	222	665	0.01
11.420	2026874	8933905	95.80
12.215	2065	29498	0.32
13.095	68598	295865	3.17
13.431	1893	10226	0.11
13.859	766	3303	0.04
14.141	258	959	0.01

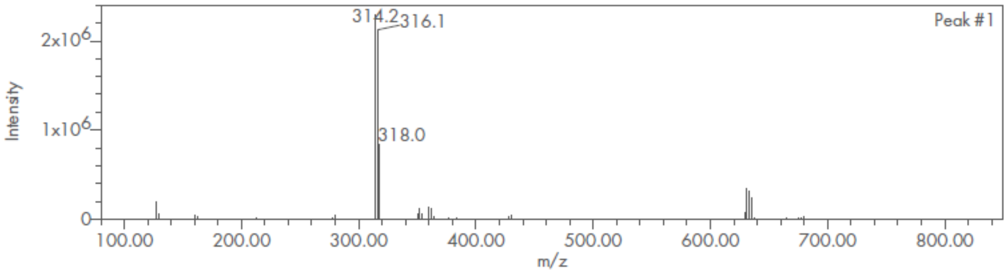

Channel description: 80.00-900.00 ES-, Centroid, CV=25 Retention time: 11.465

Figure S2. Absence of significant correlation between compounds' solubility and biochemical data. A – Distribution of thermal shift (ΔTm) values from the first screen performed with GCDH WT and DeepSCAM predictions (0, compound unlikely to aggregate at a concentration of 30 µM; 1, compound likely to aggregate at 30 µM).[32] Pearson correlation analysis (table) was performed between cLogS (or DeepSCAM prediction) and the WT thermal shift (ΔTm) values for the concentrations of compound 10, 30, and 100 µM. No statistically significant correlations were found, except for a low correlation (r=0.453) between cLogS and the ΔTm for 100 µM. **B** - Distribution of biochemical data (relative enzyme activity, tryptophan quenching derived K_B, thermal shift assay combined score) from the validation studies performed and the cLogS values. **C** – Correlation matrix for the different biochemical parameters and solubility indicators (DeepSCAM prediction and cLogS). Pearson correlation values (r) are shown on the top (black) and the respective *p* values are shown in the bottom (blue). No significant correlations were found, except for a correlation between the two solubility indicators (*r* = -0.675, *p* = 0.0002). Correlation analysis was performed using Pearson correlation matrices using GraphPad Prism 10.

A

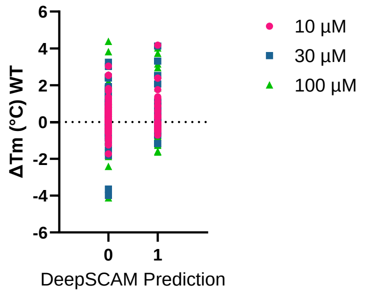

Correlation Analysis for Screening Phase I and II

	Pearson r	*p* value
DeepSCAM Prediction vs ΔTm (°C) WT (1st Screen with 10µM)	0.108	0.166
DeepSCAM Prediction vs ΔTm (°C) WT (1st Screen with 30µM)	0.062	0.429
DeepSCAM Prediction vs ΔTm (°C) WT (1st Screen with 100µM)	0.054	0.488
cLogS vs ΔTm (°C) WT (1st Screen with 10µM)	0.029	0.899
cLogS vs ΔTm (°C) WT (1st Screen with 30µM)	0.376	0.085
cLogS vs ΔTm (°C) WT (1st Screen with 100µM)	0.453	**0.030**

B

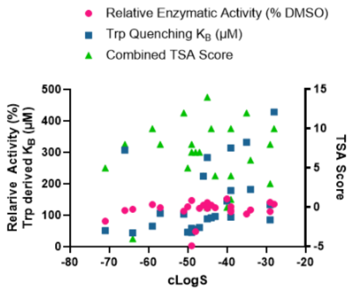

C

Correlation Matrix - Pearson correlation r (black) and respective *p* value (blue)

	ITC Kd (µM)	Scaffold	Relative Enzymatic Activity (%	Combined TSA Score	Trp Quenching Kb (µM)	log S	Deep SCAMs Prediction
ITC Kd (µM)			**-0.214**	**-0.726**	**-0.377**	**-0.600**	**0.195**
Scaffold			**-0.194**	**-0.166**	**-0.015**	**0.344**	**-0.111**
Relative Enzymatic Activity (% DMSO)	0.729	0.488		**-0.158**	**0.211**	**0.208**	**-0.343**
Combined TSA Score	0.165	0.554	0.451		**0.284**	**0.103**	**0.213**
Trp Quenching Kb (µM)	0.532	0.956	0.311	0.170		**0.376**	**-0.368**
cLogS	0.285	0.209	0.319	0.623	0.064		**-0.675**
DeepSCAMs Prediction	0.753	0.693	0.093	0.306	0.070	**0.000**	

Figure S3. Isothermal titration calorimetry (ITC) analysis of the interaction of glutaryl-CoA dehydrogenase (GCDH) variant V400M with compound A71. Heat peak intensities were plotted against time (top) and used to calculate ΔH (change in enthalpy), which was plotted against the GCDH WT/compound molar ratio (based on monomer concentration; bottom) for Kd (dissociation constant) determination. V400M showed an interaction with compound A71 with a Kd of 117 ± 91 μM.

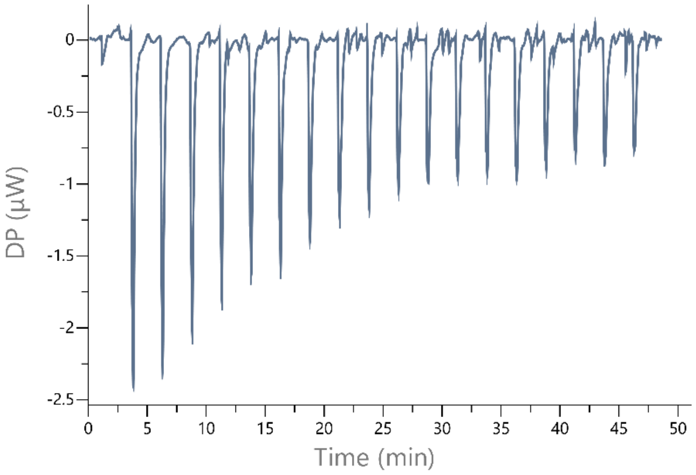

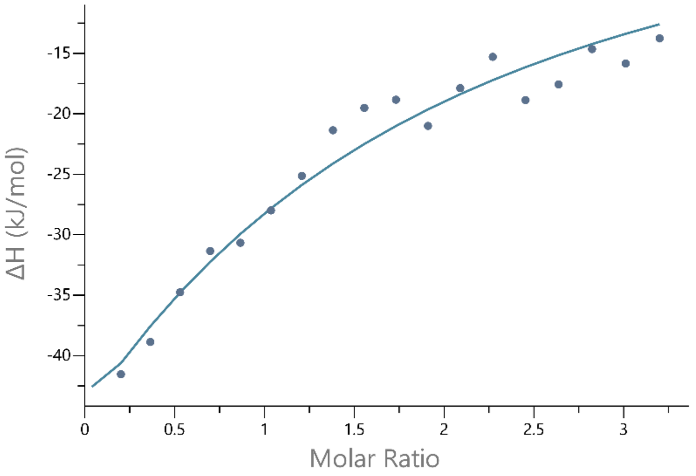

Figure S4. Dose-dependent effect on thermal stability of glutaryl-CoA dehydrogenase (GCDH) wild type (WT) and variants in the presence of compounds A49, A55, A71, B29, and B31. Thermal shift assays were run for each protein variant, WT, R88C, V400M, A433E using different compound concentrations, from 0.05 to 500 μM. A Tm (melting temperature: the temperature at which 50% of a protein is unfolded) was calculate for each concentration and the Tms were plotted against compound concentration. Tm's are shown as mean and error bars corresponding to standard deviation (n=3).

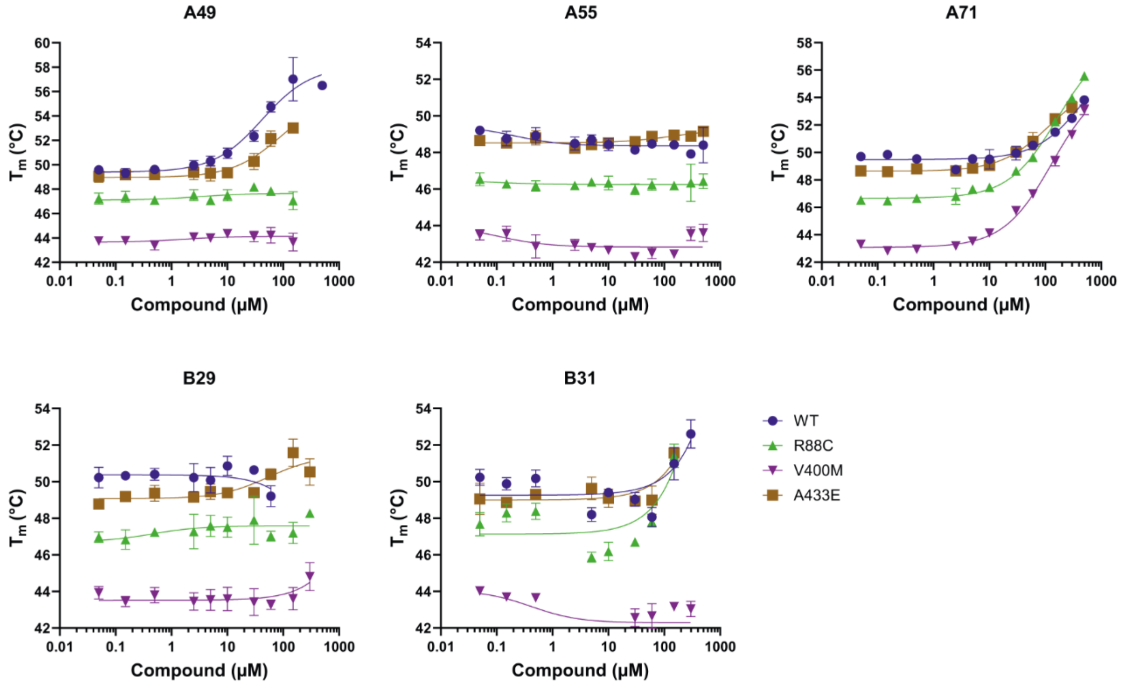

Figure S5. Dose-dependent effect on thermal stability of glutaryl-CoA dehydrogenase (GCDH) wild type (WT) and mutations mapping to the proposed allosteric binding site in the presence of lead compounds A49, A55, A71, B29, and B31. Thermal shift assays were performed for each protein variant (WT, A433E, and allosteric pocket mutant T109A, and double mutant T109A and K357A), using different compound concentrations, from 0.05 to 500 µM. Variant A433E was used as an extra control for a pocket independent mutation. A Tm (melting temperature: the temperature at which 50% of a protein is unfolded) was calculated for each concentration and the Tms were plotted against compound concentration. Tms are shown as mean and error bars corresponding to standard deviation (n=3).

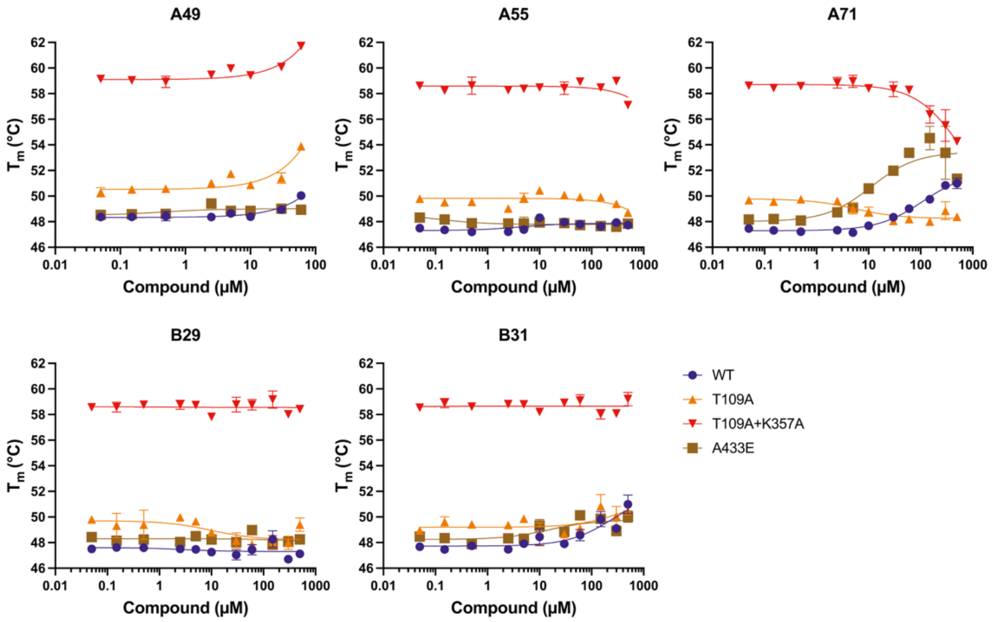

Supplement: Supplementary file 1 — jm4c00292_si_001.pdf [file jm4c00292_si_001.pdf]
